# Associations of polymorphisms in *TXNIP* and gene–environment interactions with the risk of coronary artery disease in a Chinese Han population

**DOI:** 10.1111/jcmm.12929

**Published:** 2016-07-29

**Authors:** Xue‐bin Wang, Ya‐di Han, Shuai Zhang, Ning‐hua Cui, Ze‐jin Liu, Zhu‐liang Huang, Cong Li, Fang Zheng

**Affiliations:** ^1^ Center for Gene DiagnosisZhongnan Hospital of Wuhan UniversityWuhanHubeiChina; ^2^ Department of Clinical LaboratoryChildren's Hospital of ZhengzhouZhengzhouHenanChina; ^3^ Center of Clinical LaboratoryWuhan Asia Heart HospitalWuhanHubeiChina; ^4^ Zhongshan School of MedicineSun Yat‐sen UniversityGuangzhouChina

**Keywords:** *TXNIP*SNPs, *TXNIP* expression, CAD risk, severity of coronary atherosclerosis, gene–environment interactions

## Abstract

Single nucleotide polymorphisms (SNPs) in thioredoxin‐interacting protein (*TXNIP*) gene may modulate *TXNIP* expression, then increase the risk of coronary artery disease (CAD). In a two‐stage case–control study with a total of 1818 CAD patients and 1963 controls, we genotyped three SNPs in *TXNIP* and found that the variant genotypes of SNPs rs7212 [odds ratio (OR) = 1.26, *P* = 0.001] and rs7211 (OR = 1.23, *P* = 0.005) were significantly associated with increased CAD risk under a dominant model. In haplotype analyses, compared with the reference haplotype, haplotype ‘G‐T’ had a 1.22‐fold increased risk of CAD (*P* = 0.003). We also observed the cumulative effects of SNPs rs7212 and rs7211 on CAD risk and the severity of coronary atherosclerosis. Moreover, the gene–environment interactions among the variant genotypes of SNP rs7212, smoking habit, alcohol drinking habit and history of type 2 diabetes were associated with a 3.70‐fold increased risk of CAD (*P* < 0.001). Subsequent genotype‐phenotype correlation analyses further observed the significant effects of SNP rs7212 on *TXNIP* mRNA expression, plasma TXNIP and malondialdehyde levels. Taken together, our data suggest that *TXNIP* SNPs may individually and cumulatively affect CAD risk through a possible mechanism for regulating *TXNIP* expression and gene–environment interactions.

## Introduction

Coronary artery disease (CAD), which is mediated by multiple interactions of genetic and environmental factors 1, is the leading cause of death and disability worldwide 2. The main pathogenesis of CAD is atherosclerosis, a process of accumulated deposition of lipoproteins in the coronary artery and its branches that results in impaired or absent blood supply to the heart and eventually myocardial infarction 3. Atherosclerosis is seen as a chronic inflammatory process 4 and is influenced by multiple events, such as oxidative stress caused by the excessive production of reactive oxygen species (ROS) 5. Oxidative stress can induce a series of molecular changes 6, including oxidative damage of macromolecules, proliferation and migration of vascular smooth muscle cells (VSMC), and apoptosis in the endothelial cells, all of which involve the atheroma formation 6.

Thioredoxin‐interacting protein (TXNIP), a binding protein of thioredoxin (TRX), mainly acts as an oxidative stress modulator by inhibiting TRX's antioxidant activity 7 and interacting with antioxidant transcription factors such as Nrf2 8. Moreover, molecular studies also demonstrated that TXNIP linked oxidative stress to inflammatory response through activating NLPR3 inflammasome 9 and regulating chromatin modification 10. Besides its crucial role in oxidative damage and inflammation, in epidemiological studies, TXNIP was further correlated with higher carotid intima‐media thickness 11 and abnormal glucose metabolism 12, which have been considered as a surrogate marker 13 and a traditional risk factor 14 for CAD respectively. All these findings, combined with the significant effect of a *TXNIP* polymorphism on arterial stiffness 15, support the hypothesis that single nucleotide polymorphisms (SNPs) in *TXNIP* gene may modify *TXNIP* expression and protein levels, and thus contribute to CAD risk.

Hence, in this study, we detected *TXNIP* mRNA expression, plasma TXNIP and malondialdehyde (MDA) levels, and carried out a two‐stage case–control study to evaluate the associations of three *TXNIP* SNPs with CAD risk and the severity of coronary atherosclerosis, followed by multifactor dimensionality reduction (MDR) and classification and regression tree (CART) analyses to investigate the interaction effects of *TXNIP* SNPs and traditional cardiovascular (CV) risk factors on CAD risk.

## Materials and methods

### Study population

A two‐stage case–control design was used in our study. The discovery set (Study 1) with 812 CAD patients and 957 controls was recruited from Wuhan Asia Heart Hospital between January 2011 and December 2012. In the replication set (Study 2), 1006 cases and 1006 controls were enrolled from Zhongnan Hospital of Wuhan University between May 2013 and December 2015. CAD was angiographically confirmed as stenosis of more than 50% in at least one major coronary artery or their main branches. Then, for each case, the severity of coronary atherosclerosis was assessed by vessel scores 16 and modified Gensini scores 17 (Data S1). Patients with cardiac diseases such as congenital or valvular heart diseases, coronary artery spasm and myocardial bridge, or systemic diseases such as renal or hepatic diseases, autoimmune diseases and cancers were excluded. The control groups consisted of participants without stenosis confirmed by coronary angiography (1054 controls) and healthy individuals without CV disease identified by physical examination (909 controls), and also excluded participants with the aforementioned cardiac and systemic diseases. The case and control groups from two sets were well matched for age, sex and geographical area. Data on traditional CV risk factors 14, 18 such as smoking status, alcohol drinking status and histories of hypertension, hyperlipidaemia and type 2 diabetes mellitus (T2DM) (Data S1), and clinical data such as body mass index (BMI), blood pressure, lipid and fasting plasma glucose (FPG) levels were also recorded. This study was approved by the Ethics Committees of Wuhan Asia Heart Hospital and Zhongnan Hospital of Wuhan University and followed the Declaration of Helsinki. All participants were ethnic Han Chinese and signed written informed consent.

### Selection of SNPs and genotyping

*TXNIP* gene is located on chromosome 1q21.1 and spans a relatively small genomic region of less than 4200 base pairs. According to the data from HapMap database (http://hapmap.ncbi.nlm.nih.gov/, phase1, 2&3, Hapmap‐CHB) and 1000 Genome Project (http://www.1000genomes.org/) 19, only three SNPs in *TXNIP* gene, which were rs9245 in 5′‐untranslated region (UTR) as well as rs7211 and rs7212 in 3′‐UTR, had a minor allele frequency of ≥5% in the Chinese population and were selected in our study (Table S1).

Genomic DNA was prepared from peripheral blood leucocytes using a phenol/chloroform method. SNP genotyping was conducted with high resolution melting (HRM) analyses on a LightScanner 96 system (Idaho Technology, Salt Lake City, UT, USA), as described previously 20, 21. The accuracy of genotyping was confirmed by repeated assays (Fig. S1) and direct sequencing (Fig. S2). Detailed information for HRM and sequencing analyses, such as primer sequences and PCR conditions, was summarized in Table S2.

### Reverse‐transcription quantitative PCR analysis of *TXNIP* mRNA

Total RNA of peripheral blood leucocytes was isolated using Trizol reagent (Invitrogen, Carlsbad, CA, USA), followed by elimination of DNA contamination using the RNase‐Free gDNA eraser and reverse transcription (1 μg of total RNA) using a reverse transcriptase kit (Takara Bio Inc, Kusatsu, Shiga, Japan). According to the MIQE guidelines 22, reverse‐transcription quantitative PCR (RT‐qPCR) analysis was carried out to determine *TXNIP* mRNA expression on a CFX96 Touch system (Bio‐Rad, Hercules, CA, USA) using the SYBR‐Green method (Bio‐Rad). The relative expression of *TXNIP* was normalized to the internal reference gene (*GAPDH*) and was calculated using the 2^−∆∆Cq^ method 23. Primer sequences and RT‐qPCR conditions for *TXNIP* and *GAPDH* were also presented in Table S2.

### Determination of plasma TXNIP levels

After the whole blood of each participant was centrifuged (2000 × g for 10 min. at 4°C), plasma samples were collected and stored at −80°C until use. Based on the manufacturer's instructions, the concentrations of TXNIP were detected by ELISA (TXNIP ELISA kit; Xinfan Systems, Shanghai, China), and were then quantified by a standard curve with the detection range of 20–480 pg/ml. Plasma MDA, a well‐known biomarker for oxidative damage 24, was also determined using the thiobarbituric acid reactive substances assay, as described by Girotti *et al*. 25. The coefficient of variance values for intra‐ and inter‐assays were 5.4% and 7.8% for TXNIP, and 6.5% and 8.1% for MDA respectively.

### Methylation levels at cg19693031 determined by bisulphite pyrosequencing

Bisulphite treatment of genomic DNA (1 μg) was first conducted using the EZ DNA Methylation Kit (Zymo Research, Irvine, CA, USA), followed by PCR amplification using the bisulphite‐treated DNA (~20 ng) and the PyroMark PCR kit (Qiagen, Hilden, Germany). Then, methylation levels at cg19693031 within 3′‐UTR of *TXNIP* were quantified by pyrosequencing assays using the PyroMark Q96 MD instrument (Qiagen), as described in Table S2. Non‐CPG cytosines, fully methylated and unmethylated DNA were used for quality controls. Methylation levels for each sample were calculated as the mean of two independent runs.

### MDR and CART analyses

To investigate the interaction effects of *TXNIP* SNPs and traditional CV risk factors on CAD risk, MDR and CART analyses were performed by MDR 3. 0. 2 (UPenn, Philadelphia, PA, USA) and Clementine 12.0 (SPSS Inc., Chicago, IL, USA) programs respectively. In MDR analyses 26, all possible combinations of included variables were constructed, then 100‐fold cross‐validation and 1000‐time permutation tests were conducted to assess the predictive accuracy of each model for CAD risk. The interaction model that had the maximal cross‐validation consistency (CVC) and testing accuracy as well as the most significant *P*‐value for permutation test was considered as the best predictor. Classification and regression tree analyses could find an optimal combination of genetic and environmental factors to predict CAD risk by hierarchically building a binary classification tree 27. In CART analyses, Gini index was used as the splitting criterion and the minimal terminal node (TN) size was 50 28. When the final pruned tree was constructed, the association of each TN with CAD risk was assessed by logistic regression analyses.

### Statistical analyses

For clinical data, the differences in quantitative and qualitative variables between cases and controls were assessed by the Student's *t*‐test and the Pearson chi‐squared test respectively. For each SNP, Hardy–Weinberg equilibrium (HWE) was examined by the Pearson chi‐squared test. The associations of *TXNIP* SNPs with CAD risk were evaluated by multivariable logistic regression under different genetic models after adjusting for age, sex, BMI, smoking status, alcohol drinking status and histories of hypertension, hyperlipidaemia and T2DM. When subgroup analyses were performed, the multiplicative likelihood ratio test was used to test the possible gene–environment interaction effects on CAD risk. The effects of *TXNIP* SNPs on vessel scores and modified Gensini scores were analysed by the linear‐by‐linear association chi‐squared test and the Mann–Whitney *U*‐test respectively. The differences in expression (*TXNIP* mRNA expression), plasma (TXNIP and MDA) and methylation (cg19691031) markers between cases and controls, as well as the associations of *TXNIP* SNPs with each marker were evaluated by analyses of covariance (ancova) after adjusting for covariates. The Pearson (for normal distributed data) or Spearman (for skewed data) correlation test were used to test the correlations of plasma TXNIP levels with modified Gensini scores and plasma MDA levels, as well as the effects of cg19691031 on glycated haemoglobin A1c (HbA1c) (%), FPG, and *TXNIP* mRNA expression. To correct for multiple testing, the Bonferroni correction test was conducted. All the above tests were carried out by SPSS 17.0 (SPSS Inc.) and statistical significance was set as *P* < 0.05 (two‐sided).

The gene‐based association analysis was conducted by a web‐based VEGAS program (http://gump.qimr.edu.au/VEGAS/) 29, which could combine the *P*‐values of three individual SNPs to determine the overall significance of *TXNIP* gene region after correcting for the linkage disequilibrium (LD) structure. The LD structure was constructed by Haploview 4.2 software (Broad Institute, Cambridge, MA, USA) 30. Haplotype analyses were performed by Haplo Stats 1.5.0 program (Mayo Clinic, Rochester, MN, USA) 31. This program used the expectation‐maximization algorithm to estimate haplotype frequencies, and performed the score statistics to assess the association of each haplotype with CAD risk after adjusting for covariates. Power analyses were performed by PS 3.0 program (Vanderbilt University, Nashville, TN, USA).

## Results

### Population characteristics

In two sets of our study, age and gender distributions were similar between cases and controls, while there were significant differences in BMI, blood pressure, lipid and FPG levels as well as the rates of smoking, alcohol drinking, hypertension, hyperlipidaemia and T2DM among the two groups (Table S3). The genotype frequencies of all three SNPs did not deviate from HWE in controls (*P* > 0.05, Table S1).

### Single locus analyses

In the combined population with 1818 cases and 1963 controls (Table 1 and Table S4), allelic association analyses showed that the minor alleles of SNP rs7212 (OR = 1.18, *P* = 0.008) and rs7211 (OR = 1.19, *P* = 0.006) were significantly associated with increased CAD risk. In genotypic association analyses, we also found significant associations of SNP rs7212 and rs7211 with increased CAD risk under both additive (rs7212: OR = 1.19, *P* = 0.007; rs7211: OR = 1.19, *P* = 0.006) and dominant models (rs7212: OR = 1.26, *P* = 0.001; rs7211: OR = 1.23, *P* = 0.005). All these associations remained significant after the Bonferroni correction (Table 1). Power analyses showed that the merged sample size could provide sufficient power (α = 0.05, 90.5% for rs7212; 83.2% for rs7211) to detect the associations with the dominant ORs of 1.26 and 1.23 for SNPs rs7212 and rs7211 respectively. However, there was no significant association between SNP rs9245 and CAD risk in this study. The gene‐based association analysis indicated that *TXNIP* was a susceptible gene for CAD (*P* = 0.004), and SNP rs7212 was the most susceptible locus in this gene (*P* = 0.002).

**Table 1:** Associations of TXNIP SNPs with CAD risk in 1818 CAD patients and 1963 controls

SNPs	Alleles/genotypes *N* (%)		OR (95% CI)*	*P* */*P*_BON_ †
	CAD	Controls		
rs7212				
C	2936 (80.7)	3294 (83.9)	1 (Ref)	
G	700 (19.3)	632 (16.1)	**1.18 (1.04–1.34)**	**0.008/0.024**
CC	1171 (64.4)	1387 (70.7)	1 (Ref)	
CG	594 (32.7)	520 (26.4)	**1.29 (1.11–1.50)**	**0.001/0.003**
GG	53 (2.9)	56 (2.9)	1.01 (0.68–1.51)	0.961
CG + GG	647 (356)	576 (29.3)	**1.26 (1.10–1.46)**	**0.001/0.003**
Additive			**1.19 (1.05–1.35)**	**0.007/0.021**
rs7211				
C	2944 (81.0)	3289 (83.8)	1 (Ref)	
T	692 (19.0)	637 (16.2)	**1.19 (1.05–1.34)**	**0.006/0.018**
CC	1191 (65.5)	1386 (70.6)	1 (Ref)	
CT	562 (30.9)	517 (26.3)	**1.23 (1.06–1.42)**	**0.007/0.021**
TT	65 (3.6)	60 (3.1)	1.25 (0.86–1.82)	0.239
CT + TT	627 (34.5)	577 (29.4)	**1.23 (1.07–1.42)**	**0.005/0.015**
Additive			**1.19 (1.05–1.34)**	**0.006/0.018**
rs9245				
C	2791 (76.8)	3025 (77.1)	1 (Ref)	
A	845 (23.2)	901 (22.9)	1.00 (0.90–1.12)	0.984
CC	1061 (58.4)	1156 (58.9)	1 (Ref)	
CA	669 (36.8)	713 (36.3)	1.01 (0.88–1.16)	0.874
AA	88 (4.8)	94 (4.8)	0.98 (0.71–1.34)	0.877
CA + AA	757 (41.6)	807 (41.1)	1.01 (0.88–1.15)	0.918
Additive			1.00 (0.89–1.12)	0.985

*P‐value from logistic regression after adjustment for age, sex, BMI, smoking status, alcohol drinking status and histories of hypertension, hyperlipidaemia and T2DM. ^†^Multiple testing by the Bonferroni correction, *P*‐value multiplied 3 (3 SNPs) to get a *P*_BON_ value. Bold values are statistically significant with *P* < 0.05. CAD, coronary artery disease; *N*: number; OR (95% CI): odds ratio (95% confidence interval); Ref: reference.

### Haplotype and cumulative analyses of SNPs rs7212 and rs7211

Because SNPs rs7212 and rs7211 constructed a moderate LD block (in all participants: D’ = 0.86, *r* ^2^ = 0.76, Fig. S3), haplotype analyses of these two SNPs were performed. As presented in Table 2 and Table S5, significant differences in haplotype distributions were identified between the case and control groups. Compared with the most common haplotype ‘C‐C’ (alleles in order of SNPs rs7212 and rs7211), haplotypes ‘G‐C’ (OR = 1.53, *P* = 0.003, *P*_BON_ = 0.012) and ‘G‐T’ (OR = 1.22, *P* = 0.003, *P*_BON_ = 0.012) showed significant associations with increased CAD risk. In a cumulative analysis of SNPs rs7212 and rs7211 based on a dominant model (*i.e*. risk genotypes: CC + CG for SNP rs7212; CC + CT for SNP rs7211), compared with participants without risk genotypes, individuals with one and two risk genotypes were associated with a 1.43‐fold (*P* = 0.009, *P*_BON_ = 0.036) and a 1.25‐fold (*P* = 0.003, *P*_BON_ = 0.012) increased risk of CAD respectively. When we merged individuals with risk genotypes (one or two) into one group, the cumulative effect of SNPs rs7212 and rs7211 on CAD risk became more significant (OR = 1.28, *P* < 0.001, *P*_BON_ = 0.001).

**Table 2:** Associations of haplotypes and risk genotypes of SNP rs7212 and rs7211 with CAD risk

Genotypes	CAD, *N* (%)	Controls, *N* (%)	*P*_trend_	OR (95% CI)*	*P* */*P*_BON_ †
Total no. of participants	1818	1963			
Total no. of haplotypes	3636	3926			
Haplotypes‡					
C‐C	2843 (78.2)	3221 (82.0)	**2.44 × 10** ^**−4**^	1 (Ref)	
G‐T	599 (16.5)	564 (14.4)		**1.22 (1.08–1.39)**	**0.003/0.012**
G‐C	101 (2.8)	68 (1.7)		**1.53 (1.15–2.02)**	**0.003/0.012**
C‐T	93 (2.5)	73 (1.9)		1.32 (0.90–1.85)	0.104
No. of risk genotypes					
0	1110 (61.1)	1328 (67.7)	**0.001**	1 (Ref)	
1	142 (7.8)	117 (3.0)		**1.43 (1.09–1.87)**	**0.009/0.036**
2	566 (31.1)	518 (26.3)		**1.25 (1.08–1.45)**	**0.003/0.012**
1 + 2	708 (38.9)	635 (32.3)		**1.28 (1.12–1.48)**	**<0.001/0.001**

*P‐value from logistic regression after adjustment for age, sex, BMI, smoking status, alcohol drinking status and histories of hypertension, hyperlipidaemia and T2DM. ^†^Multiple testing by the Bonferroni correction, *P*‐value multiplied 4 (4 haplotypes or four types of combined genotypes) to get a *P*_BON_ value. ^‡^Each haplotype was constructed with the order of SNPs rs7212 and rs7211. Bold values are statistically significant with *P* < 0.05. CAD: coronary artery disease; *N*: number; OR (95% CI): odds ratio (95% confidence interval); Ref: reference.

### Subgroup analyses

To further explore the potential gene–environment interactions in CAD risk, subgroup analyses were performed based on a dominant model. For SNP rs7212, after the Bonferroni correction, the associations of the variant genotypes (CC + CG) with increased CAD risk remained significant in participants who were thinner (BMI ≤25; *P*_BON_ = 0.040), smokers (*P*_BON_ = 0.004) and drinkers (*P*_BON_ = 0.001) as well as participants with T2DM (*P*_BON_ = 0.008). Moreover, the multiplicative likelihood ratio test suggested the significant interactions of SNP rs7212 with smoking status (*P*_inter_ = 0.022), alcohol drinking status (*P*_inter_ = 0.014) and history of T2DM (*P*_inter_ = 0.022; Table 3). For SNP rs7211, the associations between the variant genotypes (CC + CT) and increased CAD risk were more significant to withstand the Bonferroni correction in participants who were thinner (*P*_BON_ = 0.048) and smokers (*P*_BON_ = 0.040; Table 3). When we combined the genotypes of these two SNPs, the combined risk genotypes were consistently associated with increased CAD risk in almost all subgroups, except for non‐smokers, non‐drinkers, non‐T2DM participants and those with BMI >25 (Table S6).

**Table 3:** Stratification analyses of *TXNIP* SNPs (rs7212 and rs7211) and CAD risk under a dominant model in our study

Variables	SNP rs7212 (cases/controls, *N*)		OR (95% CI)*	*P* */*P*_BON_ †	*P*_inter_ ‡	SNP rs7211 (cases/controls, *N*)		OR (95% CI)*	*P* */*P*_BON_ †	*P*_inter_ ‡
	CC	CG + GG				CC	CT + TT			
Age, years										
≤60	580/633	329/255	1.30 (1.05–1.62)	0.018/0.072	0.817	588/627	321/261	1.26 (1.01–1.56)	0.038/0.152	0.929
>60	591/754	318/321	1.26 (1.04–1.53)	0.021/0.084		603/759	306/316	1.24 (1.02–1.51)	0.033/0.132	
Sex										
Male	625/752	372/335	1.24 (1.02–1.50)	0.024/0.096	0.686	638/749	359/338	1.19 (0.98–1.44)	0.079	0.546
Female	546/635	275/241	1.29 (1.04–1.60)	0.020/0.080		553/637	268/239	1.29 (1.04–1.60)	0.021/0.084	
BMI, kg/m^2^										
≤25	636/868	369/379	**1.28 (1.06–1.54)**	**0.010/0.040**	0.791	646/872	359/375	**1.27 (1.05–1.53)**	**0.012/0.048**	0.903
>25	535/519	278/197	1.38 (1.06–1.80)	0.018/0.072		545/514	268/202	1.36 (1.04–1.77)	0.023/0.092	
Smoking status										
Yes	374/381	265/163	**1.55 (1.21–2.00)**	**0.001/0.004**	**0.022**	397/384	242/160	**1.40 (1.08–1.80)**	**0.010/0.040**	0.234
No	797/1006	382/413	1.14 (0.96–1.36)	0.144		794/1002	385/417	1.16 (0.98–1.38)	0.093	
Drinking status										
Yes	317/330	263/153	**1.66 (1.26–2.18)**	**<0.001/0.001**	**0.014**	356/332	224/151	1.28 (0.97–1.68)	0.082	0.778
No	854/1057	384/423	1.13 (0.95–1.34)	0.159		835/1054	403/426	1.21 (1.02–1.43)	0.028/0.112	
Hypertension										
Yes	693/547	387/232	1.28 (1.04–1.56)	0.019/0.076	0.889	702/541	378/238	1.22 (0.99–1.49)	0.059	0.883
No	478/840	260/344	1.24 (1.01–1.51)	0.040/0.160		489/845	249/339	1.24 (1.01–1.52)	0.038/0.152	
T2DM										
Yes	335/356	260/163	**1.53 (1.18–1.99)**	**0.002/0.008**	**0.022**	371/366	224/153	1.36 (1.04–1.77)	0.024/0.096	0.321
No	836/1031	387/413	1.15 (0.97–1.36)	0.116		820/1020	403/424	1.18 (0.99–1.40)	0.061	
Hyperlipidaemia										
Yes	334/326	194/129	1.38 (1.04–1.83)	0.026/0.104	0.359	339/322	189/133	1.32 (1.00–1.75)	0.050/0.200	0.438
No	837/1061	453/447	1.22 (1.03–1.44)	0.020/0.080		852/1064	438/444	1.19 (1.01–1.41)	0.040/0.160	

*P‐value from logistic regression after adjustment for age, sex, BMI, smoking status, alcohol drinking status and histories of hypertension, hyperlipidaemia and T2DM. ^†^Multiple testing by the Bonferroni correction, *P*‐value multiplied 4 (3 SNPs + 1 combined risk genotypes of SNPs rs7212 and rs7211) to get a *P*_BON_ value. ^‡^ *P*‐value from the multiplicative likelihood ratio test to assess the multiplicative interaction effects of *TXNIP* SNPs and selected variables on CAD risk. Bold values are statistically significant after the Bonferroni correction. *N*: number; OR (95% CI): odds ratio (95% confidence interval); BMI: body mass index; T2DM: type 2 diabetes mellitus.

### MDR and CART analyses

**Figure 1:**
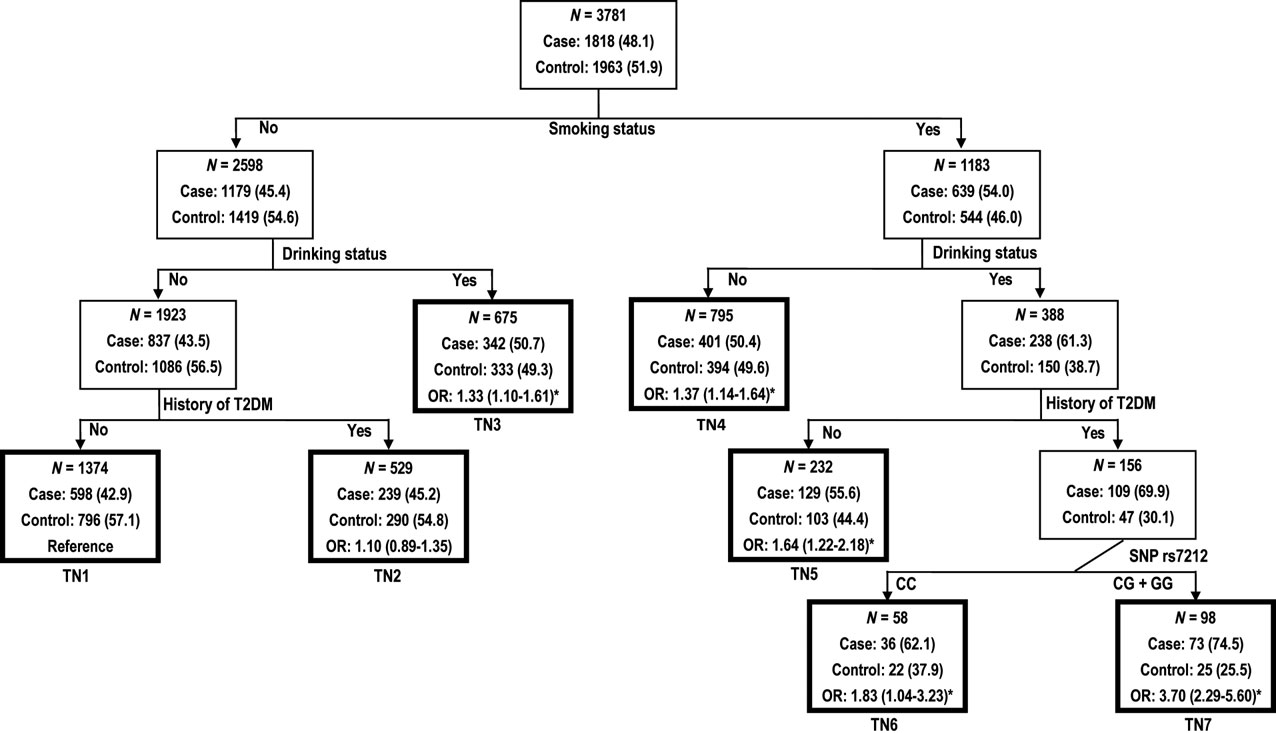
Classification and regression tree for smoking status, alcohol drinking status, history of T2DM and SNP rs7212 in all participants of our study. Terminal nodes (TN) are thick bordered. ORs and 95% CIs were calculated by logistic regression after adjusting for age, sex, BMI, smoking status, alcohol drinking status and histories of hypertension, hyperlipidaemia and T2DM, *P < 0.05.

According to the results of the multiplicative likelihood ratio test, data on SNP rs7212, smoking status, alcohol drinking status and history of T2DM were included in MDR and CART analyses to further scrutinize the best interaction models. As summarized in Table 4, in MDR analyses, the four‐factor model including all variables had the maximal CVC of 100/100 and the optimal testing accuracy of 0.6215 as well as the most significant *P*‐value (*P* < 0.0001) for permutation test, and was therefore considered as the best predictor for CAD risk. In CART analyses (Fig. 1), ‘smoking status’, ‘alcohol drinking status’, ‘history of T2DM’ and ‘SNP rs7212’ variables were selected as the primary, secondary, tertiary and terminal split nodes, respectively, indicating that smoking habit exerted the strongest effect on CAD risk, followed by drinking habit, history of T2DM and the variant genotypes (CC + CG) of SNP rs7212. Furthermore, compared with the reference group, participants with smoking and drinking habits, T2DM and the CC + CG variant genotypes had a 3.70‐fold (*P* < 0.001) increased risk of CAD, suggesting the existence of gene–environment interactions. When the combined genotypes of SNPs rs7212 and rs7211 were included in MDR and CART analyses, similar gene–environment interactions were also found between the combined genotypes and three traditional risk factors (Table 4 and Fig. S4).

**Table 4:** The best models to predict CAD risk by MDR analyses

No. of risk factors	Best interaction models	CVC*	Testing accuracy (%)†	*P* for permutation test‡
1	Smoking status	84/100	0.5585	0.0356
2	Smoking status, alcohol drinking status	94/100	0.5873	0.0001
3	Smoking status, alcohol drinking status, history of T2DM	100/100	0.6011	<0.0001
4	**Smoking status, alcohol drinking status, history of T2DM, SNP rs7212 (CC/CG + GG)**	100/100	0.6215	<0.0001
4	**Smoking status, alcohol drinking status, history of T2DM, no. of risk genotypes (0/1 + 2)**	100/100	0.6283	<0.0001

*CVC means the number of times that a given combination of factors is identified in each testing set (a total of 100 times). ^†^Testing accuracy (%) is the percentage of participants for whom a correct prediction is made. ^‡^The permutation test was carried out to repeat the MDR analyses 1000 times and to calculate the CVC and testing accuracy of each n‐factor model. Bold values indicate the models that have the maximal CVC and the optimal testing accuracy as well as the most significant *P*‐value for permutation test. T2DM: type 2 diabetes mellitus; CVC: cross‐validation consistency.

### Associations of *TXNIP* SNPs with the severity of coronary atherosclerosis

As summarized in Table 5, in single locus analyses, only the variant genotypes (CC + CG) of SNP rs7212 were associated with higher modified Gensini scores (*P* = 0.011). In a cumulative analysis of SNPs rs7212 and SNP rs7211, CAD patients with 1‐2 risk genotypes had higher modified Gensini scores than those without risk genotypes (*P* = 0.008). Moreover, when the severity of coronary atherosclerosis was assessed by vessel scores, we further observed a dose–response effect of the increasing number of risk genotypes on higher vessel scores (*P* = 0.025).

**Table 5:** Associations of *TXNIP* SNPs (rs7212 and rs7211) with the severity of coronary atherosclerosis in 1818 CAD patients

The severity of coronary atherosclerosis	SNP rs7212			SNP rs7211			No. of risk genotypes		
	CC, *N* (%)	CG + GG, *N* (%)	*P*_trend_	CC, *N* (%)	CT + TT, *N* (%)	*P*_trend_	0, *N* (%)	1 + 2, *N* (%)	*P*_trend_
Vessel score‐1	426 (66.4)	216 (33.6)	0.112	441 (68.7)	201 (31.3)	0.107	**417 (65.0)**	**225 (35.0)**	**0.025**
Vessel score‐2	395 (64.6)	216 (35.4)		386 (63.2)	225 (36.8)		**361 (59.1)**	**250 (40.9)**	
Vessel score‐3	350 (61.9)	215 (38.1)		364 (64.4)	201 (35.6)		**332 (58.8)**	**233 (41.2)**	
Modified Gensini score^a^	**29.0 (18.0–70.5)**	**35.0 (19.5–79.5)**	**0.011**	30.0 (18.0–74.0)	33.0 (19.5–74.0)	0.155	**29.0 (18.0–72.0)**	**35.0 (20.0–75.0)**	**0.008**

a Modified Gensini scores were expressed as median (interquartile range) because of the skewed distributions. Bold values indicate statistically significant with *P* < 0.05.

### Correlations of *TXNIP* SNPs with *TXNIP* mRNA expression, plasma TXNIP and MDA levels

We detected *TXNIP* mRNA expression, plasma TXNIP and MDA levels in 240 participants (120 CAD patients and 120 controls) randomly selected from two sets. These participants had similar genetic and clinical characteristics as compared to the overall population (Table S7). After adjusting for covariates, ancova models showed that CAD patients had higher levels of *TXNI*P mRNA expression (1.20 ± 0.28 *versus* 1.06 ± 0.28, *P* = 0.001), plasma TXNIP (289.3 ± 23.0 pg/ml *versus* 274.1 ± 23.0 pg/ml, *P* < 0.001) and MDA (1.76 ± 0.35 μmol/l *versus* 1.61 ± 0.32 μmol/l, *P* = 0.011) than the control group (Table S7).

**Figure 2:**
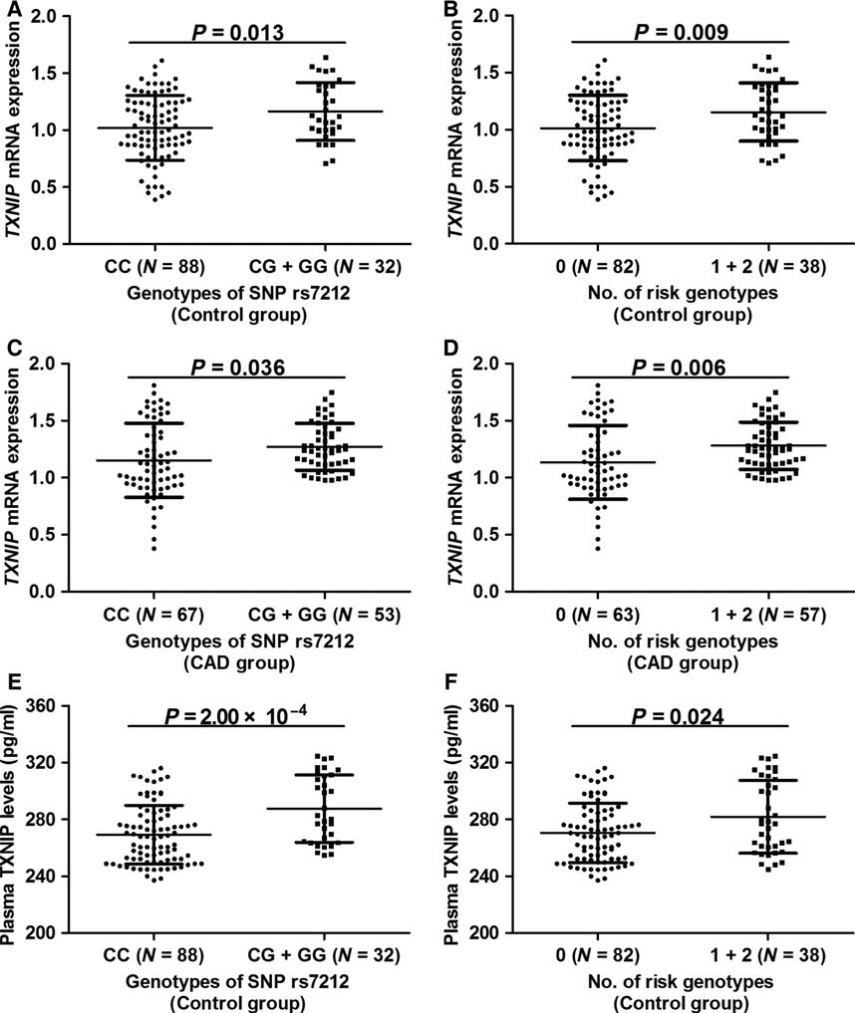
Associations of *TXNIP* SNPs with *TXNIP* mRNA expression and plasma TXNIP levels. (**A**) Associations of SNP rs7212 with *TXNIP* mRNA expression in the control group. (**B**) Associations of no. of risk genotypes with *TXNIP* mRNA expression in the control group. (**C**) Associations of SNP rs7212 with *TXNIP* mRNA expression in the CAD group. (**D**) Associations of no. of risk genotypes with *TXNIP* mRNA expression in the CAD group. (**E**) Associations of SNP rs7212 with plasma TXNIP levels in the control group. (**F**) Associations of no. of risk genotypes with plasma TXNIP levels in the control group. ancova models were used to assess statistical significance. Data were expressed as mean ± S.D.

In healthy controls (Fig. 2 and Table S8), the variant genotypes (CC + CG) of SNP rs7212 were significantly correlated with increased levels of *TXNIP* mRNA expression (*P* = 0.013) and plasma TXNIP (*P* < 0.001). In CAD patients, participants with the CG + GG genotypes had higher levels of *TXNIP* mRNA expression compared with the CC carriers (*P* = 0.036). Similarly, in a cumulative analysis of SNPs rs7212 and rs7211, significant correlations were also found between the risk genotypes and higher levels of *TXNIP* mRNA expression in both the case (*P* = 0.006) and control (*P* = 0.009) groups, and between the risk genotypes and higher plasma TXNIP levels only in the case group (*P* = 0.024). Moreover, the Spearman correlation test also found that the plasma TXNIP levels were positively correlated with modified Gensini scores in CAD patients (*r* = 0.220, *P* = 0.016).

Subsequently, the CG + GG genotypes of SNP rs7212, the risk genotypes of SNP rs7212+ rs7211, and increased plasma TXNIP levels were consistently associated with higher MDA levels in both cases and controls (Fig. S5 and Table S7), suggesting the effects of *TXNIP* SNPs on oxidative damage.

### Correlations between *TXNIP* SNPs and cg19693031

Cg19693031, a CPG site within 3′‐UTR of *TXNIP*, was recently reported by two epigenome‐wide association studies to be associated with T2DM, HbA1c (%) and FPG 32, 33. To test the possible interactions between cg19693031 and *TXNIP* SNPs, 48 participants (24 CAD patients *versus* 24 controls) with data on HbA1c (%), FPG, *TXNIP* mRNA expression and plasma TXNIP were selected to determine methylation levels at cg19693031. As presented in Figure S6, methylation levels at cg19693031 were inversely correlated with HbA1c (%) (*r* = −0.318, *P* = 0.027), FPG (*r* = −0.299, *P* = 0.039), *TXNIP* mRNA expression (*r* = −0.383, *P* = 0.007) and CAD risk (*P* = 0.032), but were not associated with SNP rs7212 and rs7211.

## Discussion

This study, for the first time, reported the significant associations of *TXNIP* SNPs with increased CAD risk. Several lines of evidence reinforced this finding. Firstly, using a two‐stage case–control design with a total of 1818 CAD patients and 1963 controls, we found that *TXNIP* SNPs were significantly associated with increased CAD risk and the severity of coronary atherosclerosis in both single locus and cumulative analyses. Secondly, by performing subgroup, MDR and CART analyses, we further validated the interaction effects of SNP rs7212 and three traditional CV risk factors on CAD risk. Finally, subsequent genotype‐phenotype correlation analyses supported that SNP rs7212 might be functional by affecting *TXNIP* expression and protein levels.

It is widely accepted that oxidative inflammatory response plays a vital role in the development of CAD. *TXNIP*, an up‐regulated gene of oxidative stress, encodes a TXNIP that inhibits the antioxidant activity of TRX protein 7 and Nrf2 transcription factor 8, actives NLPR3 inflammasome 9, and thus links oxidative stress to inflammation 9. Recently, several studies further showed that TXNIP could induce the inflammatory response in endothelial cells and VSMC by increasing leucocyte adhesion 34 and regulating the expression of adhesion molecules 35 and anti‐inflammatory transcription factors (such as Kruppel‐like factor 2) 34, and therefore involve the atherogenesis 35. In the current study, we also found a significant correlation between SNP rs7212 and plasma MDA levels, suggesting the effects of *TXNIP* SNPs on oxidative damage. Moreover, by combing the significance of all three *TXNIP* SNPs, the gene‐based association analysis also suggested that *TXNIP* gene was associated with increased CAD risk. All these findings together provide biological evidence that *TXNIP* is a susceptible gene for CAD.

SNPs rs7212 and rs7211, which constructed a moderate LD pattern in 3′‐UTR of *TXNIP*, were associated with CAD risk in single locus, haplotype and cumulative analyses of our study. Recently, a cross‐sectional study from Brazil 36 has reported that the minor allele of SNP rs7212 was correlated with increased *TXNIP* expression levels in VSMC. This study validated this correlation in leucocytes and further found a significant association between the variant genotypes of SNP rs7212 and increased levels of plasma TXNIP. Notably, two recent epigenome‐wide association studies 32, 33 identified that methylation levels at cg19693031 within 3′‐UTR of *TXNIP* were inversely associated with T2DM, HbA1c (%) and FPG. This latest evidence, combined with the physical proximity between cg19693031 and SNP rs7212 (<800 bases), prompted us to test whether SNP rs7212 interacted with cg19693031 to regulate *TXNIP* expression. However, although we found the significant associations of cg19693031 with HbA1c (%), FPG and *TXNIP* mRNA expression, SNP rs7212 and rs7211 were not correlated with methylation levels at cg19693031. These results are consistent with the previous report that methylation levels at cg19693031 were not influenced by 42 SNPs (not including SNPs rs7212 and rs7211) adjacent to cg19693031 (<50 kb) 37, suggesting that genetic and epigenetic variations in 3′‐UTR of *TXNIP* may independently affect *TXNIP* expression.

Generally, the potential functions of a causal SNP in 3′‐UTR of a gene are to change mRNA stability and translation efficiency 38 either by mutating the binding sites of microRNAs (miRNAs) and RNA‐binding proteins 39, or by altering the stem‐loop structures 40. The location of SNP rs7212 is in the distal one‐third of the 3′‐UTR, and is only 300 bases away from the poly A tail. This region contains a lot of *cis*‐acting elements, such as AU‐rich elements and U‐rich region. Therefore, from the standpoint of physical location, this SNP has the potential to influence the stability of *TXNIP* mRNA. Moreover, by searching a publicly available SNP‐miRNA interactions database (miRNASNP 2.0, http://bioinfo.life.hust.edu.cn/miRNASNP2/index.php) 41, we further found that the minor allele G of SNP rs7212 disrupted the binding sites of several miRNAs, including hsa‐miR‐92 and hsa‐miR‐296, which have been reported to protect against CAD 42. This evidence reinforces the possibility that SNP rs7212 may regulate *TXNIP* expression by interacting with miRNAs to further influence mRNA stability. However, for SNP rs7211, the position of this locus is relatively far (about 1100 bases) from the poly A tail and does not contain any known elements. Besides, our study also failed to find any significant association of SNP rs7211 with *TXNIP* expression and protein levels in the Chinese Han population. Considering that SNPs rs7211 and rs7212 are located in the same LD block, it is reasonable to hypothesize that SNP rs7211 may well be a marker of SNP rs7212, but not a causal one. In summary, taking all the above evidence together, we suggest that SNP rs7212 may contribute to CAD risk by affecting *TXNIP* expression and protein levels.

In this study, by performing subgroup, MDR and CART analyses, we also found the interaction effects of SNP rs7212 and three traditional CV risk factors (*i.e*. smoking status, alcohol drinking status and history of T2DM) on CAD risk. From a biological perspective, it is well known that smoking and drinking habits can cause excessive ROS production, either directly by inducing peroxidation, or indirectly through depletion of endogenous antioxidants (such as vitamin C) 43, 44. Accordingly, their indirect impact on ROS generation is similar to the role of TXNIP protein in oxidative damage and CAD risk. Moreover, for history of T2DM, besides the known impact of dysglycaemia on CAD risk 45, numerous studies have also reported the effect of TXNIP protein on abnormal glucose metabolism 12, 46 and the significant associations between *TXNIP* SNPs and DM risk 36. All these findings, combined with the significant correlations of SNP rs7212 with *TXNIP* expression and protein levels, suggest that the gene–environment interactions among SNP rs7212, smoking status, alcohol drinking status and history of T2DM may greatly increase CAD risk. In our CART analyses, we observed that compared with the reference group, participants with smoking and drinking habits, T2DM and the CC + CG variant genotypes had a 3.70‐fold increased risk of CAD. This result partially supports the above view and needs to be further explained by functional studies.

In this study, we calculated modified Gensini scores and vessels scores, and found the significant correlations of SNP rs7212 and plasma TXNIP levels with the severity of coronary atherosclerosis in a Chinese population. These results are supported by the report that TXNIP‐ApoE double knockout mice exhibited a dramatic reduction in atherosclerotic lesion size at aorta 35 and the evidence that SNP rs7212 was significantly associated with arterial stiffness in a Brazilian population 15. All these findings validate the crucial role of SNP rs7212 and TXNIP protein in the development of atherosclerosis, and the detailed mechanism needs to be elucidated in future studies.

Some limitations of our study also merit consideration. First, although we have matched for age, sex and geographical location between cases and controls, the selection bias might be inevitable because of the inherent drawback of retrospective study. Second, in our study, we only genotyped common SNPs in *TXNIP*. Fine‐mapping studies are encouraged to find low‐frequency variants in this gene. Finally, although we have collected data on several CV risk factors, other risk factors might also involve gene–environment interactions.

In conclusion, our study shows that *TXNIP* SNPs may individually and cumulatively contribute to CAD risk by affecting *TXNIP* expression and protein levels as well as by interacting with smoking status, alcohol drinking status and history of T2DM. Future studies are needed to replicate these results and explore the underlying mechanism.

## Conflict of interest

The authors declare no competing financial interests.

## Supporting information


**Figure S1** HRM plots for different genotypes of three SNPs.
**Figure S2** Direct sequencing analyses for different genotypes of three SNPs.
**Figure S3** Analysis of the LD structure.
**Figure S4** Classification and regression tree for smoking status, alcohol drinking status, history of T2DM and the combined risk genotypes (SNPs rs7212+ rs7211) in all participants of our study.
**Figure S5** Associations of plasma MDA levels with SNP rs7212, No. of risk genotypes and plasma TXNIP levels.
**Figure S6** Associations of methylation levels at cg19693031 with HbA1c (%), FPG, *TXNIP* mRNA expression, CAD risk and *TXNIP* SNPs.
**Table S1** Characteristics of 3 SNPs in *TXNIP* gene.
**Table S2** Primer details and PCR conditions for HRM, direct sequencing and RT‐qPCR analyses in our study.
**Table S3** Clinical characteristics of participants in our study.
**Table S4** Associations of *TXNIP* SNPs with CAD risk in two sets of our study.
**Table S5** Associations of haplotypes and risk genotypes of SNP rs7212 and rs7211 with CAD risk in two sets of our study.
**Table S6** Stratification analyses of the combined risk genotypes (SNP rs7212+ rs7211) and CAD risk in our study.
**Table S7** Comparative analyses of clinical and genetic characteristics between the randomly selected participants and the whole samples.
**Table S8** Associations of *TXNIP* SNPs with *TXNIP* mRNA expression, plasma TXNIP and MDA levels.
**Data S1** Supplementary materials and methods.
