# Supplementary material for: Associations of polymorphisms in TXNIP and gene–environment interactions with the risk of coronary artery disease in a Chinese Han population
Source: J Cell Mol Med. 2016 Jul 29;20(12):2362–73. doi: 10.1111/jcmm.12929 (PMC5134401; doi:10.1111/jcmm.12929)

**Associations of Polymorphisms in *TXNIP* and Gene-Environment Interactions with the Risk of Coronary Artery Disease in a Chinese Han Population**

Xue-bin Wang ^a, #^, Ya-di Han ^a, #^, Shuai-Zhang ^a^, Ning-hua Cui ^b^, Ze-jin Liu ^c^, Zhu-liang Huang ^a^, Cong Li^d,^ * and Fang Zheng ^a,^ *

^a^ Center for Gene Diagnosis, Zhongnan Hospital of Wuhan University, Wuhan, Hubei, China

^b^ Department of Clinical Laboratory, Children's Hospital of Zhengzhou, Zhengzhou, Henan, China

^c^ Center of Clinical Laboratory, Wuhan Asia Heart Hospital, Wuhan, Hubei, China

^d^ Zhongshan School of Medicine, Sun Yat-sen University, Guangzhou, China

* Corresponding author : Fang Zheng

Email: [zhengfang@whu.edu.cn](mailto:zhengfang@whu.edu.cn)

Telephone number: 862767813233

Correspondence may also be addressed to Cong Li, email: licong28@mail.sysu.edu.cn

^#^ These authors contributed equally to this work and should be considered as co-first authors.

**Supplementary materials and methods**

**Criterion of vessel scores and modified Gensini scores**

In the current study, the coronary angiograms were independently evaluated by two experienced angiographers who were blinded to the clinical and genetic data of each subject. Vessel scores ranged from 1 to 3 according to the number of vessels having ≥ 50% stenosis. In the modified Gensini scoring system, angiographic stenosis of each coronary segment was first scored according to the degree of luminal narrowing: 1 for 0-25% stenosis, 2 for 26-50%, 4 for 51-75%, 8 for 76-90%, 16 for 91-99% and 32 for 100%. Then a multiplier was assigned to each segment depending on the functional significance of the area supplied by that segment: 5 for main left coronary artery (MLCA), 2.5 for proximal left anterior descending coronary artery (LAD) and proximal left circumflex branch, 1.5 for mid-segment of LAD, 0.5 for second diagonal branch and posterolateral branch, and 1 for other branches. Finally, the weighted scores for each segment were added to give modified Gensini scores. As acute coronary occlusion usually occurs in a previous angiographically non-critical lesion, modified Gensini scoring system scores acute total occlusion as a non-critical lesion (0-5 score) instead of true chronic total occlusion (32-172 score).The κ values for inter-observer and intra-observer variability were 0.95 and 0.94 for vessel scores and 0.93, 0.90 for modified Gensini scores, respectively.

**Definition of clinical characteristics**

Individuals who smoked ≥ 100 cigarettes in their lifetime were defined as “smokers”, which included ever smokers and current smokers. An ever smoker was a person who had quit smoking at least 1 year prior to interview. Subjects with alcohol consumption at least once a week for ≥ 1 year were defined as “alcohol drinkers”. One drink was defined as 375 ml of beer (13.6 g of ethanol), 118 ml of wine (11.7 g of ethanol), or 30 ml of western or Chinese hard liquor (10.9 g of ethanol). Hypertension was defined as ongoing therapy for hypertension, systolic blood pressure (SBP) of ≥ 140mmHg or diastolic blood pressure (DBP) of ≥ 90mmHg. Type 2 diabetes mellitus (T2DM) was defined as ongoing therapy for diabetes or fasting plasma glucose (FPG) levels of ≥ 7.0 mmol/L, or with plasma glucose levels of ≥ 11.1mmol/L, or a 2-h plasma glucose level of ≥ 11.1mmol/L during an oral glucose tolerance test. Hyperlipidemia was defined as hypercholesterolemia (serum TC > 5.72 mmol/L), high levels of LDL-c (> 3.1 mmol/L), low levels of HDL-c (< 0.9 mmol/L), hypertriglyceridemia (serum TG > 1.70 mmol/L).

**Supplementary Table**

| **Table S1** Characteristics of 3 SNPs in *TXNIP* gene. | | | | | | | | |
| --- | --- | --- | --- | --- | --- | --- | --- | --- |
| SNP | Position * | Location | Minor/Major allele | MAF (%) † | |  | P (HWE) ‡ | |
|  |  |  |  | Study 1 | Study 2 |  | Study 1 | Study 2 |
| rs7212 | Chr1: 145992816 | 3’-UTR | G/C | 16.1 | 16.1 |  | 0.801 | 0.341 |
| rs7211 | Chr1: 145993449 | 3’-UTR | T/C | 16.1 | 16.3 |  | 0.212 | 0.475 |
| rs9245 | Chr1: 145996488 | 5’-UTR | A/C | 23.1 | 22.8 |  | 0.272 | 0.548 |
| Chr, chromosome; UTR, untranslated region; MAF, minor allele frequency; HWE, Hardy-Weinberg equilibrium.  * Information for chromosome position was based on NCBI genome build 38.2.  † MAF was calculated from the genotype data in our controls.  ‡ The P value for HWE was calculated from the genotype data in our controls. | | | | | | | | |

| **Table S2** Primer details and PCR conditions for HRM, sequencing, RT-qPCR and pyrosequencing analyses in our study. | | | | |
| --- | --- | --- | --- | --- |
| Method | Variable | Primers | Length (bp) | Ann. Temp (°C) |
| HRM * | rs7212 | Forward: GAGGAACTCTTGATCAAGATGC | 46 | 56 |
|  |  | Reverse: TGGAGGTTCTGAACACAGG |  |  |
|  | rs7211 | Forward: ATTGTGATAGGAACTTTGGACC | 53 | 54 |
|  |  | Reverse: TGGCTCTTCTCCACATGATAC |  |  |
|  | rs9245 | Forward: GGGAAAGAAGGCTTTTTCTCTGA | 51 | 59 |
|  |  | Reverse: TATACGCCGCTGGTTACAC |  |  |
| Sequencing † | rs7212 | Forward: GCTGCTCTACGGTCATCT | 487 | 57 |
|  |  | Reverse: CTGCCCTCTAGTTTCTCAT |  |  |
|  | rs7211 | Forward: CCTTAGCCTCTGACTTCC | 513 | 53 |
|  |  | Reverse: AAGCAAAGATGACCGTAGA |  |  |
|  | rs9245 | Forward: GGGAGAAGACGCTCAACA | 573 | 54 |
|  |  | Reverse: GAGTTAGAAATGACGGTGGA |  |  |
| RT-qPCR ‡ | *TXNIP* | Forward: GCAATCATATTATCTCAGGGAC | 133 | 57 |
|  |  | Reverse: GGAACGCTAACATAGATCAGTAA |  |  |
|  | *GAPDH* | Forward: GAAGGTGAAGGTCGGAGTC | 226 | 57 |
|  |  | Reverse: GAAGATGGTGATGGGATTTC |  |  |
| Pyrosequencing § | cg19693031 | Forward (biotinated): TAGGGGTGTTTGTTGGATGGGTTTAA | 258 | 56 |
|  |  | Reverse: ATTTTAACCACAATTTCCCTTTTCATCTT |  |  |
|  |  | Sequencing: GGGTTAGGTAAAAATGG |  |  |
| HRM, High Resolution Melt; Ann. Temp., annealing temperature; RT-qPCR, reverse-transcription quantitative PCR.  * PCR reaction for HRM was performed in a volume of 10 μL containing 25 ng of genomic DNA, 5 pmol of each primer, 2 mmol of dNTPs, 2 μL of 10×PCR buffer with 1.5 mmol/L MgCl_2_, 1 unit of Taq polymerase and 1 μL of LC green. The cycling conditions for HRM analyses are 95 °C for 5 min followed by 45 cycles of 20 s at 95 °C, 20 s at the respective annealing temperatures and 30 s at 72 °C, and final extensions of 30 s at 95 °C and 30 s at 28 °C for heteroduplex formation.  † The cycling conditions for sequencing are 95 °C for 5 min, 35 cycles of 95 °C for 30 s, 30s at the respective annealing temperatures and 45 s at 72 °C.  ‡ RT-qPCR analysis was performed in a final volume of 20 μL reaction mixture containing 10 μL of 1 X SYBR Green Master mix (Bio-rad), 5 pmol of each primer and 50 ng of cDNA products. The cycling conditions for RT-qPCR are 95 °C for 5 min and 40 cycles of 95 °C for 15 s and 57 °C elongation for 45s.  § After bisulfite treatment of genomic DNA, PCR amplification was performed in a volume of 25 μL containing about 20 ng of bisulfite-treated DNA, 0.2 μM of each (forward and reverse) primer, and 12 μL of the PCR Master Mix. Then, biotin-labelled PCR products were captured by Streptavidin Sepharose bead, followed by washing (with 70% ethanol for 5s), alkaline-denaturing (with denaturation buffer for 15s) and rewashing (with washing buffer for 15s) in the Vacuum Prep Tool (Qiagen). Subsequently, the single-strand DNA was annealed with 1.6 μM of sequencing primers at 80°C for 2 min. After pyrosequencing analyses (10 μL of single-strand DNA) using the Pyromark 96 MD instrument, methylation status at cg19693031 was analyzed by Pyro Q CG software. | | | | |

| **Table S3** Clinical characteristics of participants in our study. | | | | | | | | | | | |
| --- | --- | --- | --- | --- | --- | --- | --- | --- | --- | --- | --- |
| Variables | Discovery set (Study 1) | | |  | Replication set (Study 2) | | |  | Merged set (Study 1 + Study 2) | | |
|  | CAD (N = 812) | Controls (N = 957) | P * |  | CAD (N = 1006) | Controls (N = 1006) | P * |  | CAD (N = 1818) | Controls (N = 1963) | P * |
| Age, years | 62.7 ± 9.8 | 62.5 ± 10.6 | 0.703 |  | 63.0 ± 9.6 | 62.5 ± 10.9 | 0.318 |  | 62.9 ± 9.7 | 62.5 ± 10.8 | 0.309 |
| Male, n (%) | 460 (56.7) | 524 (54.8) | 0.424 |  | 537 (53.4) | 563 (56.0) | 0.244 |  | 997 (54.8) | 1087 (55.4) | 0.742 |
| BMI, kg/m^2^ | 25.0 ± 4.1 | 24.1 ± 2.2 | < 0.001 |  | 25.2 ± 4.2 | 24.1 ± 2.2 | < 0.001 |  | 25.1 ± 4.2 | 24.1 ± 2.2 | < 0.001 |
| Smoking, n (%) | 280 (34.5) | 253 (26.4) | < 0.001 |  | 359 (35.7) | 291 (28.9) | 0.001 |  | 639 (35.1) | 544 (27.7) | < 0.001 |
| Alcohol drinking, n (%) | 244 (30.0) | 230 (24.0) | 0.004 |  | 336 (33.4) | 253 (25.1) | < 0.001 |  | 580 (31.9) | 483 (24.6) | < 0.001 |
| Hypertension, n (%) | 528 (65.0) | 378 (39.5) | < 0.001 |  | 552 (54.9) | 401 (39.9) | < 0.001 |  | 1080 (59.4) | 779 (39.7) | < 0.001 |
| T2DM, n (%) | 266 (32.8) | 254 (26.5) | 0.004 |  | 329 (32.7) | 265 (26.3) | 0.002 |  | 595 (32.7) | 519 (26.4) | < 0.001 |
| Hyperlipidemia, n (%) | 234 (28.8) | 224 (23.4) | 0.010 |  | 294 (29.2) | 231 (23.0) | 0.001 |  | 528 (29.0) | 455 (23.2) | < 0.001 |
| SBP, mmHG | 147.4 ± 24.7 | 130.5 ± 33.5 | < 0.001 |  | 149.9 ± 36.1 | 133.9 ± 33.8 | < 0.001 |  | 148.8 ± 31.5 | 132.3 ± 33.7 | < 0.001 |
| DBP, mmHG | 90.4 ± 17.7 | 82.2 ± 11.1 | < 0.001 |  | 91.2 ± 18.2 | 82.6 ± 12.6 | < 0.001 |  | 90.9 ± 18.0 | 82.4 ± 11.9 | < 0.001 |
| FPG, mmol/L | 5.73 ± 1.63 | 5.31 ± 1.72 | < 0.001 |  | 5.69 ± 1.81 | 5.36 ± 1.61 | < 0.001 |  | 5.71 ± 1.73 | 5.34 ± 1.66 | < 0.001 |
| TC, mmol/L | 5.01 ± 1.03 | 4.78 ± 0.85 | < 0.001 |  | 5.04 ± 0.99 | 4.83 ± 0.98 | < 0.001 |  | 5.03 ± 1.01 | 4.80 ± 0.92 | < 0.001 |
| TG, mmol/L | 1.63 ± 0.90 | 1.36 ± 0.77 | < 0.001 |  | 1.64 ± 0.87 | 1.42 ± 0.79 | < 0.001 |  | 1.63 ± 0.88 | 1.39 ± 0.78 | < 0.001 |
| LDL-c, mmol/L | 3.04 ± 0.88 | 2.77 ± 0.71 | < 0.001 |  | 2.91 ± 0.94 | 2.77 ± 0.78 | < 0.001 |  | 2.97 ± 0.92 | 2.77 ± 0.75 | < 0.001 |
| HDL-c, mmol/L | 1.02 ± 0.16 | 1.25 ± 0.21 | < 0.001 |  | 1.01 ± 0.17 | 1.25 ± 0.21 | < 0.001 |  | 1.01 ± 0.16 | 1.25 ± 0.21 | < 0.001 |
| Vessel score |  |  |  |  |  |  |  |  |  |  |  |
| 1 | 293 (36.0) | - |  |  | 349 (34.7) | - |  |  | 642 (35.3) | - |  |
| 2 | 292 (36.0) | - |  |  | 319 (31.7) | - |  |  | 611 (33.6) | - |  |
| 3 | 227 (28.0) | - |  |  | 338 (33.6) | - |  |  | 565 (31.1) | - |  |
| Modified Gensini score | 30.0 (18.0-69.0) | - |  |  | 33.0 (18.0-76.5) | - |  |  | 30.5 (18.0-74.0) | - |  |
| CAD, coronary artery disease; BMI, body mass index; T2DM, type 2 diabetes mellitus; SBP, systolic blood pressure; DBP, diastolic blood pressure; FPG, fasting plasma glucose; TC, total cholesterol; TG, triglyceride; LDL-c, low-density lipoprotein cholesterol; HDL-c, high-density lipoprotein cholesterol. * For continuous variables, normally distributed data were expressed as mean ± standard deviation (SD), while skewed data were described as median (interquartile range). For categorical, data were expressed as frequency counts. | | | | | | | | | | | |

| **Table S4** Associations of *TXNIP* SNPs with CAD risk in two sets of our study. | | | | | | | | | |
| --- | --- | --- | --- | --- | --- | --- | --- | --- | --- |
| SNPs (genotypes) | Discovery set (812 cases vs 957 controls) | | | |  | Replication set (1006 cases vs 1006 controls) | | | |
|  | CAD  N (%) | Controls  N (%) | OR (95% CI) * | P * |  | CAD  N (%) | Controls  N (%) | OR (95% CI) * | P * |
| rs7212 |  |  |  |  |  |  |  |  |  |
| C | 1323 (81.5) | 1605 (83.9) | 1 (Ref) |  |  | 1613 (80.2) | 1689 (83.9) | 1 (Ref) |  |
| G | 301 (18.5) | 309 (16.1) | 1.16 (0.97-1.40) | 0.105 |  | 399 (19.8) | 323 (16.1) | **1.20 (1.02-1.42)** | **0.031** |
| CC | 527 (64.9) | 674 (70.4) | 1 (Ref) |  |  | 644 (64.0) | 713 (70.9) | 1 (Ref) |  |
| CG | 269 (33.1) | 257 (26.9) | **1.32 (1.06-1.64)** | **0.012** |  | 325 (32.3) | 263 (26.1) | **1.28 (1.05-1.57)** | **0.017** |
| GG | 16 (2.0) | 26 (2.7) | 0.76 (0.39-1.49) | 0.427 |  | 37 (3.7) | 30 (3.0) | 1.18 (0.71-1.97) | 0.531 |
| CG + GG | 285 (35.1) | 283 (29.6) | **1.27 (1.03-1.57)** | **0.027** |  | 362 (36.0) | 293 (29.1) | **1.27 (1.04-1.54)** | **0.017** |
| Additive |  |  | 1.17 (0.97-1.42) | 0.096 |  |  |  | **1.21 (1.02-1.43)** | **0.030** |
| rs7211 |  |  |  |  |  |  |  |  |  |
| C | 1323 (81.5) | 1606 (83.9) | 1 (Ref) |  |  | 1621 (80.6) | 1683 (83.7) | 1 (Ref) |  |
| T | 301 (18.5) | 329 (16.1) | 1.19 (0.99-1.43) | 0.064 |  | 391 (19.4) | 329 (16.3) | **1.20 (1.01-1.41)** | **0.037** |
| CC | 535 (65.9) | 679 (71.0) | 1 (Ref) |  |  | 656 (65.2) | 707 (70.3) | 1 (Ref) |  |
| CT | 253 (31.2) | 248 (25.9) | **1.27 (1.02-1.59)** | **0.031** |  | 309 (30.7) | 269 (26.7) | 1.19 (0.98-1.46) | 0.085 |
| TT | 24 (2.9) | 30 (3.1) | 1.10 (0.62-1.96) | 0.751 |  | 41 (4.1) | 30 (3.0) | 1.41 (0.85-2.32) | 0.180 |
| CT + TT | 277 (34.1) | 278 (29.0) | **1.26 (1.02-1.55)** | **0.036** |  | 350 (34.8) | 299 (29.7) | **1.22 (1.00-1.48)** | **0.049** |
| Additive |  |  | 1.19 (0.99-1.43) | 0.064 |  |  |  | **1.19 (1.01-1.41)** | **0.039** |
| rs9245 |  |  |  |  |  |  |  |  |  |
| C | 1246 (76.7) | 1472 (76.9) | 1 (Ref) |  |  | 1545 (76.8) | 1553 (77.2) | 1 (Ref) |  |
| A | 378 (23.3) | 442 (23.1) | 1.02 (0.86-1.20) | 0.838 |  | 467 (23.2) | 459 (22.8) | 1.00 (0.86-1.16) | 0.964 |
| CC | 477 (58.7) | 560 (58.5) | 1 (Ref) |  |  | 584 (58.0) | 596 (59.2) | 1 (Ref) |  |
| CA | 292 (36.0) | 352 (36.8) | 0.97 (0.79-1.20) | 0.790 |  | 377 (37.5) | 361 (35.9) | 1.05 (0.87-1.27) | 0.607 |
| AA | 43 (5.3) | 45 (4.7) | 1.16 (0.74-1.83) | 0.519 |  | 45 (4.5) | 49 (4.9) | 0.85 (0.55-1.33) | 0.481 |
| CA + AA | 335 (41.3) | 397 (41.5) | 0.99 (0.81-1.21) | 0.949 |  | 422 (42.0) | 410 (40.8) | 1.03 (0.86-1.24) | 0.773 |
| Additive |  |  | 1.02 (0.86-1.20) | 0.836 |  |  |  | 1.00 (0.85-1.16) | 0.963 |
| CAD, coronary artery disease; N, number; OR (95% CI), odds ratio (95% confidence interval); Ref, reference.  * P value from logistic regression after adjustment for age, sex, BMI, smoking status, alcohol drinking status and histories of hypertension, hyperlipidemia and T2DM.  † Multiple testing by the Bonferroni correction, P-value multiplied 3 (3 SNPs) to get a P_BON_ value.  Bold values are statistically significant with P < 0.05. | | | | | | | | | |

| **Table S5** Associations of haplotypes and risk genotypes of SNP rs7212 and rs7211 with CAD risk in two sets of our study. | | | | | | | | | | | |
| --- | --- | --- | --- | --- | --- | --- | --- | --- | --- | --- | --- |
| Genotypes | Discovery set (Study 1) | | | | |  | Replication set (Study 2) | | | | |
|  | CAD, N (%) | Controls, N (%) | P _trend_ | OR (95%CI) * | P * |  | CAD, N (%) | Controls, N (%) | P _trend_ | OR (95%CI) * | P * |
| Total No. of subjects | 812 | 957 |  |  |  |  | 1006 | 1006 |  |  |  |
| Total No. of haplotypes | 1624 | 1914 |  |  |  |  | 2012 | 2012 |  |  |  |
| Haplotypes † |  |  |  |  |  |  |  |  |  |  |  |
| C-C | 1273 (78.3) | 1570 (82.0) | **0.049** | 1 (Ref) |  |  | 1570 (78.0) | 1651 (82.0) | **0.003** |  |  |
| G-T | 271 (16.7) | 273 (14.3) |  | **1.22 (1.02-1.47)** | **0.031** |  | 328 (16.3) | 291 (14.5) |  | **1.25 (1.05-1.49)** | **0.013** |
| G-C | 40 (2.5) | 36 (1.9) |  | 1.37 (0.87-2.16) | 0.174 |  | 61 (3.1) | 32 (1.6) |  | **1.70 (1.16-2.50)** | **0.007** |
| C-T | 40 (2.5) | 35 (1.8) |  | 1.41 (0.89-2.23) | 0.141 |  | 53 (2.6) | 38 (1.9) |  | 1.32 (0.92-1.88) | 0.124 |
| No. of risk genotypes |  |  |  |  |  |  |  |  |  |  |  |
| 0 | 501 (61.7) | 648 (67.7) | **0.049** | 1 (Ref) |  |  | 609 (60.5) | 680 (67.6) | **0.023** | 1 (Ref) |  |
| 1 | 60 (7.4) | 57 (6.0) |  | 1.38 (0.92-2.07) | 0.116 |  | 82 (8.2) | 60 (6.0) |  | **1.48 (1.03-2.13)** | **0.033** |
| 2 | 251 (30.9) | 252 (26.3) |  | **1.27 (1.02-1.59)** | **0.033** |  | 315 (31.3) | 266 (26.4) |  | **1.24 (1.01-1.53)** | **0.036** |
| 1 + 2 | 311 (38.3) | 309 (32.3) |  | **1.29 (1.05-1.59)** | **0.015** |  | 397 (39.5) | 326 (32.4) |  | 1.29 (1.07-1.56) | 0.009 |
| CAD, coronary artery disease; N, number; OR (95%CI), odds ratio (95% confidence interval); Ref, reference.  * P value from logistic regression after adjustment for age, sex, BMI, smoking status, alcohol drinking status and histories of hypertension, hyperlipidemia and T2DM.  † Each haplotype was constructed with the order of SNPs rs7212 and rs7211.  Bold values are statistically significant with P < 0.05. | | | | | | | | | | | |

| **Table S6** Stratification analyses of the combined risk genotypes (SNP rs7212 + rs7211) and CAD risk in our study. | | | | | |
| --- | --- | --- | --- | --- | --- |
| Variables | No. of risk genotypes (cases/controls, N) | | OR (95%CI) * | P */P_BON_ † | P_inter_ ‡ |
|  | 0 | 1 + 2 |  |  |  |
| Age, years |  |  |  |  |  |
| ≤ 60 | 546/605 | 363/283 | **1.33 (1.08-1.65)** | **0.008/0.032** | 0.804 |
| > 60 | 564/723 | 345/352 | **1.28 (1.05-1.54)** | **0.009/0.036** |  |
| Sex |  |  |  |  |  |
| Male | 589/716 | 408/371 | **1.27 (1.05-1.52)** | **0.009/0.036** | 0.711 |
| Female | 521/612 | 300/264 | **1.31 (1.06-1.62)** | **0.012/0.048** |  |
| BMI, kg/m^2^ |  |  |  |  |  |
| ≤ 25 | 597/836 | 408/411 | **1.37 (1.14-1.64)** | **0.001/0.004** | 0.616 |
| > 25 | 513/492 | 300/224 | 1.33 (1.02-1.72) | 0.033/0.132 |  |
| Smoking status |  |  |  |  |  |
| Yes | 356/365 | 283/179 | **1.53 (1.20-1.97)** | **0.001/0.004** | **0.047** |
| No | 754/963 | 425/456 | 1.18 (0.99-1.40) | 0.060 |  |
| Drinking status |  |  |  |  |  |
| Yes | 307/318 | 273/165 | **1.60 (1.22-2.09)** | **0.001/0.004** | **0.046** |
| No | 803/1010 | 435/470 | 1.18 (0.99-1.39) | 0.053 |  |
| Hypertension |  |  |  |  |  |
| Yes | 657/524 | 423/255 | **1.29 (1.06-1.57)** | **0.012/0.048** | 0.943 |
| No | 453/804 | 285/380 | **1.27 (1.04-1.54)** | **0.012/0.048** |  |
| T2DM |  |  |  |  |  |
| Yes | 323/345 | 272/174 | **1.54 (1.19-1.99)** | **0.001/0.004** | **0.044** |
| No | 787/983 | 436/461 | 1.18 (0.99-1.39) | 0.055 |  |
| Hyperlipidemia |  |  |  |  |  |
| Yes | 316/311 | 212/144 | **1.41 (1.06-1.84)** | **0.011/0.044** | 0.352 |
| No | 794/1017 | 496/491 | **1.24 (1.05-1.46)** | **0.010/0.040** |  |
| N, number; OR (95%CI), odds ratio (95% confidence interval); BMI: body mass index; T2DM, type 2 diabetes mellitus.  * P value from logistic regression after adjustment for age, sex, BMI, smoking status, alcohol drinking status and histories of hypertension, hyperlipidemia and T2DM.  † Multiple testing by the Bonferroni correction, P-value multiplied 4 (3 SNPs + 1 combined genotypes of SNPs rs7212 and rs7211) to get a P_BON_ value.  ‡ P value from the multiplicative likelihood ratio test to assess the multiplicative interaction effects of *TXNIP* SNPs and selected variables on CAD risk. Bold values are statistically significant after the Bonferroni correction. | | | | | |

| **Table S7** Comparative analyses of clinical and genetic characteristics between the randomly selected subjects and the whole samples. | | | | | | | |
| --- | --- | --- | --- | --- | --- | --- | --- |
| Variables * | Control | | |  | CAD | | |
|  | Selected subjects (N = 120) | Whole samples (N = 1963) | P † |  | Selected subjects (N = 120) | Whole samples (N = 1818) | P † |
| Age, (year) | 63.2 ± 10.8 | 62.5 ± 10.8 | 0.519 |  | 64.0 ± 10.5 | 62.9 ± 9.7 | 0.208 |
| Male, n (%) | 67 (55.8) | 1084 (55.4) | 0.922 |  | 62 (51.7) | 997 (54.8) | 0.499 |
| BMI, kg/m^2^ | 24.0 ± 2.2 | 24.1 ± 2.2 | 0.656 |  | 25.7 ± 4.4 | 25.1 ± 4.2 | 0.115 |
| Smoking, n (%) | 32 (26.7) | 544 (27.7) | 0.804 |  | 49 (40.8) | 639 (35.1) | 0.208 |
| Drinking, n (%) | 29 (24.2) | 483 (24.6) | 0.914 |  | 41 (34.2) | 580 (31.9) | 0.607 |
| Hypertension, n (%) | 45 (37.5) | 779 (39.7) | 0.635 |  | 76 (63.3) | 1080 (59.4) | 0.396 |
| T2DM, n (%) | 32 (26.7) | 519 (26.4) | 0.956 |  | 40 (33.3) | 595 (32.7) | 0.891 |
| Hyperlipidemia, n (%) | 30 (25.0) | 455 (23.2) | 0.647 |  | 34 (28.3) | 528 (29.0) | 0.868 |
| Rs7212, n (%)‡ |  |  | 0.531 |  |  |  | 0.072 |
| CC | 88 (73.3) | 1387 (70.7) |  |  | 67 (55.8) | 1171 (64.4) |  |
| CG + GG | 32 (26.7) | 576 (29.3) |  |  | 53 (44.2) | 647 (35.6) |  |
| Rs7211, n (%)‡ |  |  | 0.804 |  |  |  | 0.502 |
| CC | 86 (71.7) | 1386 (70.6) |  |  | 75 (62.5) | 1191 (65.5) |  |
| CT + TT | 34 (28.3) | 577 (29.4) |  |  | 45 (37.5) | 627 (34.5) |  |
| Risk genotypes, n (%) |  |  | 0.877 |  |  |  | 0.078 |
| 0 | 82 (68.3) | 1328 (67.7) |  |  | 63 (52.5) | 1110 (61.1) |  |
| 1 + 2 | 38 (31.7) | 635 (32.3) |  |  | 57 (47.5) | 708 (38.9) |  |
| Modified Gensini score |  |  |  |  | 26.5 (18.0-86.5) | 30.5 (18-74.0) | 0.809 |
| Vessel score, n (%) |  |  |  |  |  |  | 0.202 |
| 1 |  |  |  |  | 49 (40.8) | 642 (35.3) |  |
| 2 |  |  |  |  | 31 (25.8) | 611 (33.6) |  |
| 3 |  |  |  |  | 40 (33.3) | 565(31.1) |  |
| N, number; CAD, coronary artery disease; BMI, body mass index; T2DM, type 2 diabetes mellitus.  * For continuous variables, normally distributed data were expressed as mean ± SD, while skewed data were described as median (interquartile range). For categorical, data were expressed as frequency counts.  † The Pearson χ^2^ test and the student t-test (or the Mann-Whitney U test) were used to test for categorical variables and continuous variables, respectively.  ‡ For SNPs rs7212 and rs7211, the GG and TT carriers were relatively rare in our randomly selected subjects, so we presented the results of comparative analyses under a dominant model. | | | | | | | |

| **Table S8** Associations of *TXNIP* SNPs with *TXNIP* mRNA expression, plasma TXNIP and MDA levels. | | | | | | | | | | | | | | | | | |
| --- | --- | --- | --- | --- | --- | --- | --- | --- | --- | --- | --- | --- | --- | --- | --- | --- | --- |
| SNPs | *TXNIP* mRNA expression † | | | | |  | Plasma TXNIP levels (pg/mL) † | | | | |  | Plasma MDA levels (μmol/L) † | | | | |
|  | Control | |  | CAD | |  | Control | |  | CAD | |  | Control | |  | CAD | |
|  | N | Mean ± SD |  | N | Mean ± SD |  | N | Mean ± SD |  | N | Mean ± SD |  | N | Mean ± SD |  | N | Mean ± SD |
| Total | 120 | 1.06 ± 0.28 |  | 120 | 1.20 ± 0.28 ^*^ |  | 120 | 274.1 ± 23.0 |  | 120 | 289.3 ± 23.0 ^*^ |  | 120 | 1.61 ± 0.32 |  | 120 | 1.76 ± 0.35 ^*^ |
| SNP rs7212 |  |  |  |  |  |  |  |  |  |  |  |  |  |  |  |  |  |
| CC | 88 | 1.02 ± 0.29 |  | 67 | 1.15 ± 0.32 |  | 88 | 269.2 ± 20.8 |  | 67 | 287.4 ± 23.2 |  | 88 | 1.58 ± 0.32 |  | 67 | 1.66 ± 0.32 |
| CG | 27 | 1.18 ± 0.25 ^**^ |  | 43 | 1.24 ± 0.20 |  | 27 | 286.1 ± 23.5 ^**^ |  | 43 | 293.3 ± 21.8 |  | 27 | 1.68 ± 0.31 |  | 43 | 1.87 ± 0.34 ^**^ |
| GG | 5 | 1.14 ± 0.21 |  | 10 | 1.40 ± 0.19 ^**^ |  | 5 | 296.9 ± 25.9 ^**^ |  | 10 | 284.7 ± 26.5 |  | 5 | 1.87 ± 0.23 |  | 10 | 1.94 ± 0.34 ^**^ |
| CG + GG | 32 | 1.17 ± 0.24 ^**^ |  | 53 | 1.27 ± 0.20 ^**^ |  | 32 | 287.7 ± 23.8 ^**^ |  | 53 | 291.7 ± 22.8 |  | 32 | 1.71 ± 0.30 ^**^ |  | 53 | 1.88 ± 0.34 ^**^ |
| SNP rs7211 |  |  |  |  |  |  |  |  |  |  |  |  |  |  |  |  |  |
| CC | 86 | 1.03 ± 0.29 |  | 75 | 1.17 ± 0.32 |  | 86 | 272.6 ± 22.4 |  | 75 | 288.2 ± 23.3 |  | 86 | 1.59 ± 0.33 |  | 75 | 1.72 ± 0.34 |
| CT | 29 | 1.15 ± 0.24 |  | 35 | 1.22 ± 0.20 |  | 29 | 276.4 ± 23.3 |  | 35 | 293.9 ± 22.3 |  | 29 | 1.68 ± 0.30 |  | 35 | 1.80 ± 0.37 |
| TT | 5 | 1.00 ± 0.21 |  | 10 | 1.39 ± 0.18 |  | 5 | 287.2 ± 31.0 |  | 10 | 281.1 ± 22.7 |  | 5 | 1.68 ± 0.30 |  | 10 | 1.86 ± 0.32 |
| CT + TT | 34 | 1.13 ± 0.24 |  | 45 | 1.26 ± 0.20 |  | 34 | 278.0 ± 24.3 |  | 45 | 291.1 ± 22.8 |  | 34 | 1.68 ± 0.30 |  | 45 | 1.81 ± 0.35 |
| Risk genotypes (N) |  |  |  |  |  |  |  |  |  |  |  |  |  |  |  |  |  |
| 0 | 82 | 1.01 ± 0.29 |  | 63 | 1.13 ± 0.32 |  | 82 | 270.5 ± 20.8 |  | 63 | 287.1 ± 23.6 |  | 82 | 1.57 ± 0.32 |  | 63 | 1.66 ± 0.32 |
| 1 | 10 | 1.24 ± 0.28 ^**^ |  | 16 | 1.38 ± 0.20 ^**^ |  | 10 | 276.2 ± 33.4 |  | 16 | 293.4 ± 20.4 |  | 10 | 1.86 ± 0.30 ^**^ |  | 16 | 1.98 ± 0.28 ^**^ |
| 2 | 28 | 1.13 ± 0.24 |  | 41 | 1.24 ± 0.20 |  | 28 | 283.9 ± 22.8 ^**^ |  | 41 | 291.0 ± 23.3 |  | 28 | 1.66 ± 0.29 |  | 41 | 1.83 ± 0.36 ^**^ |
| 1 + 2 | 38 | 1.16 ± 0.29 ^**^ |  | 57 | 1.28 ± 0.21 ^**^ |  | 38 | 281.9 ± 25.7 ^**^ |  | 57 | 291.7 ± 22.4 |  | 38 | 1.72 ± 0.30 ^**^ |  | 57 | 1.87 ± 0.34 ^**^ |
| CAD, coronary artery disease; N, number; SD, standard deviation.  ^*^ P < 0.05, in the comparisons between CAD patients and healthy controls. ^**^ P < 0.05, in the comparisons between different genotypes of SNP rs7212 and rs7211 as well as in the comparisons between different numbers of risk genotypes (SNPs rs7212 + rs7211).  † Data were expressed as mean ± SD. | | | | | | | | | | | | | | | | | |


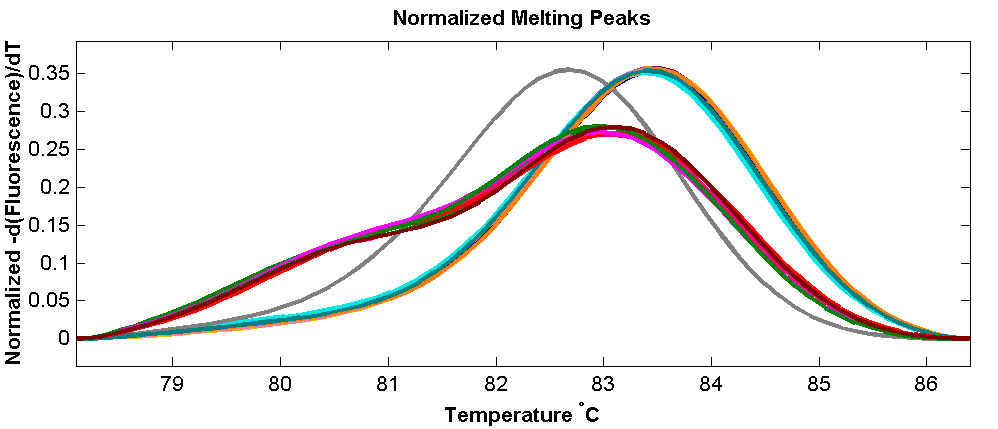

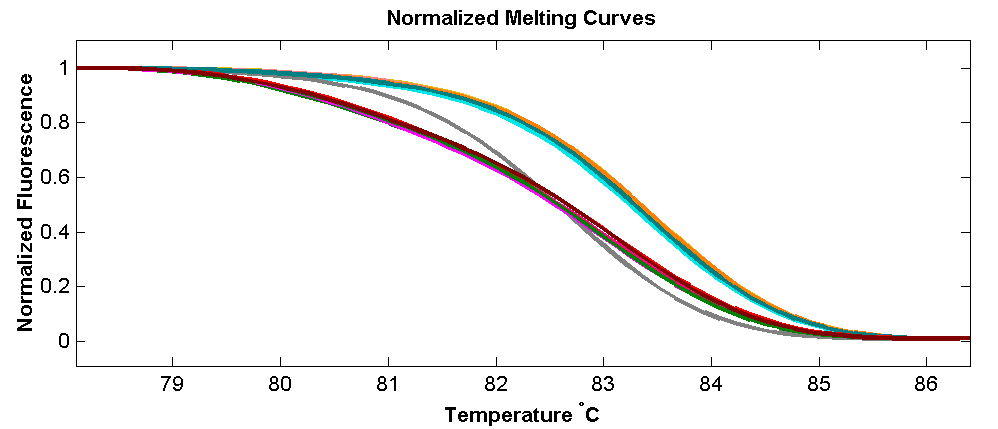


GG

CC

CG

GG

CC

CG

**a**


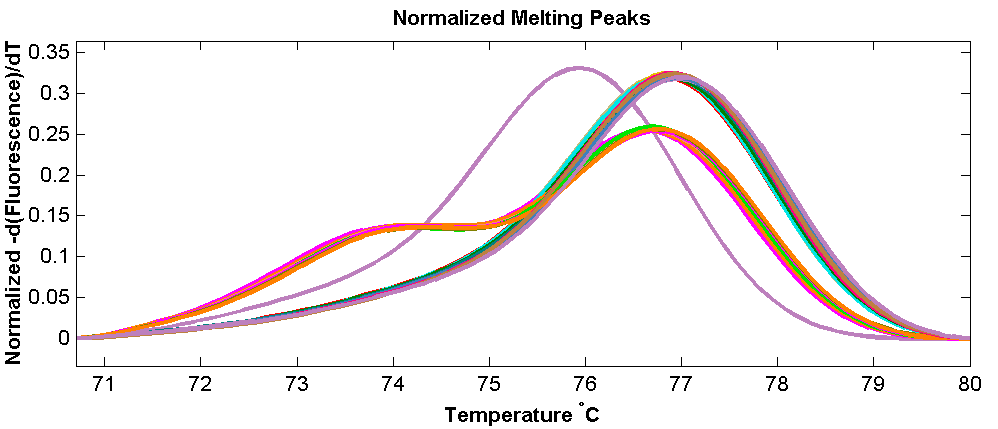

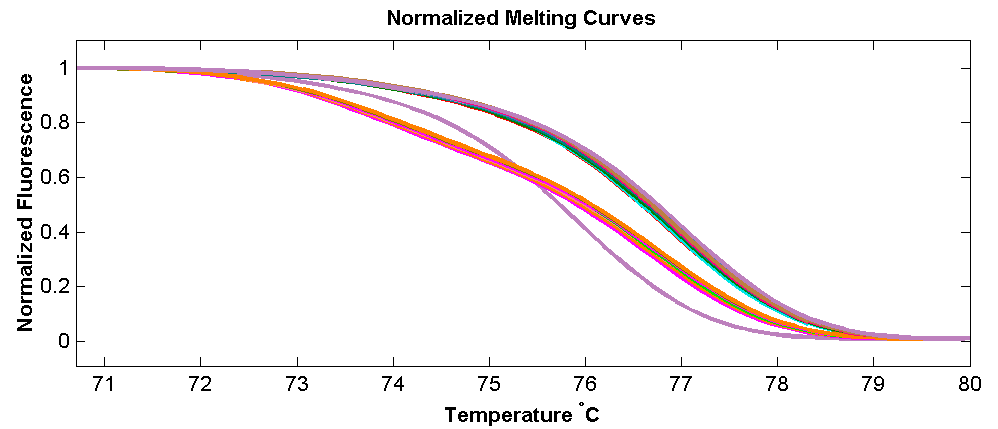


TT

CT

CC

TT

CT

CC

**b**

**c**


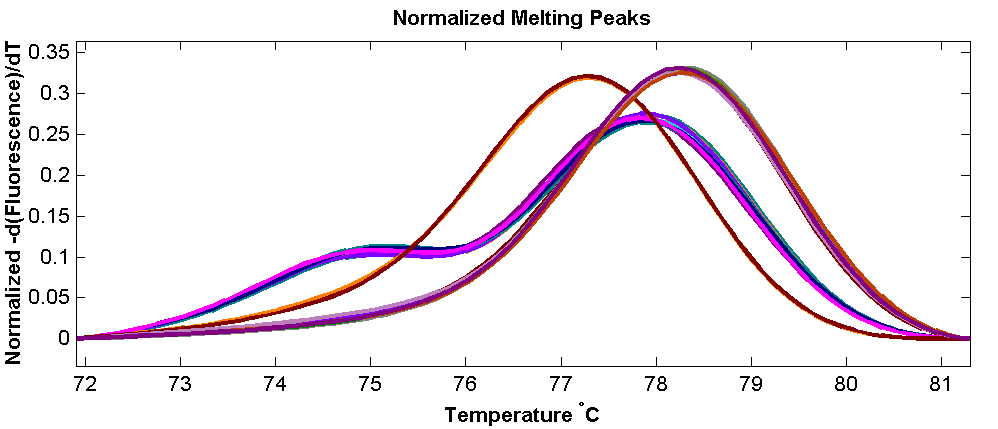


AA

CA

CC


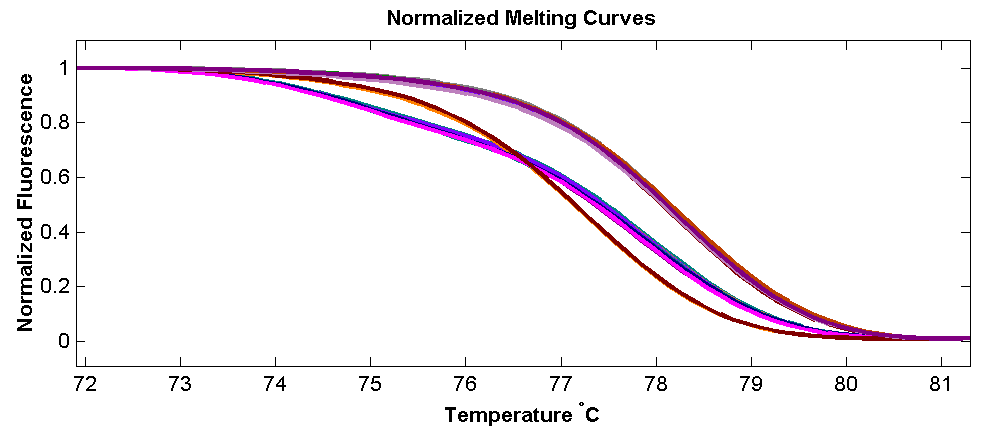


AA

CA

CC

**Figure S1 HRM plots for different genotypes of three SNPs.** The normalized melting peaks are given in the left column, and the normalized melting curves are given in the right column. Arrows indicate the genotypes. Heterozygous samples are identified by a change in melting curve shape, and different homozygotes are distinguished by melting temperature (Tm) shifts. The representative HRM plots of SNPs rs7212, rs7211 and rs9245are shown in a, b, and c, respectively.

**Supplementary Figure**


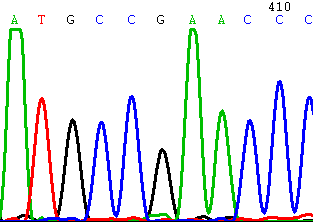

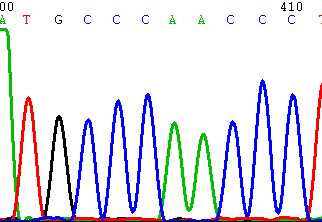

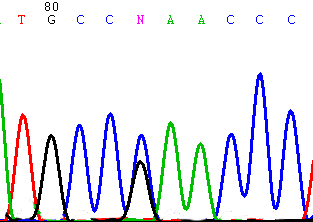


**a**


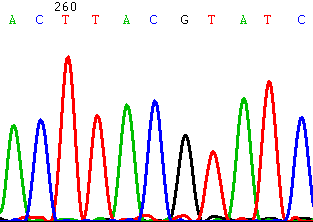

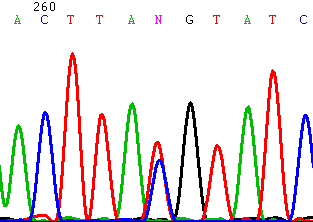

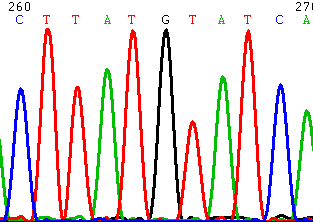


**b**


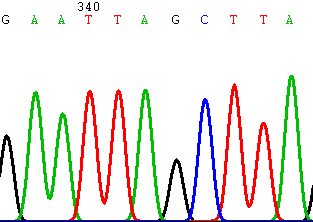

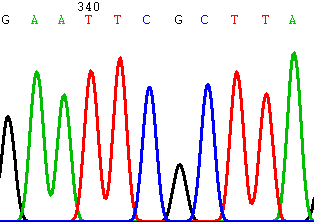

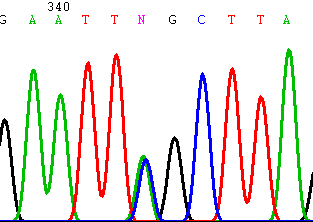


**c**

**Figure S2 Direct sequencing analyses for different genotypes of three SNPs.** The three genotypes of SNPs rs7212 (C >G), rs7211 (C>T) and rs9245 (C >A) are shown in A, B, and C, respectively.


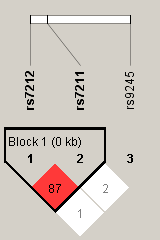

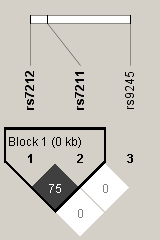


**a**

**b**

**Figure S3 Analysis of the LD structure.** The LD structure between SNPs rs7212, rs7211 and rs9245 was calculated based on genotyping data from all subjects of our study. The pairwise correlation between three SNPs was measured as D’ (a) and r^2^ (b), respectively.

**Figure S4 Classification and regression tree for smoking status, alcohol drinking status, history of T2DM and the combined risk genotypes (SNPs rs7212 + rs7211) in all subjects of our study.** Terminal nodes (TN) are thick bordered. ORs and 95% CIs were calculated by logistic regression after adjusting for age, sex, BMI, smoking status, alcohol drinking status and histories of hypertension, hyperlipidemia and T2DM. *, P < 0.05 (two-tailed).

**Smoking status**

**N = 1183**

**Case: 639 (54.0)**

**Control: 544 (46.0)**

**TN7**

**TN6**

**N = 2598**

**Case: 1179 (45.4)**

**Control: 1419 (54.6)**

**N = 1923**

**Case: 837 (43.5)**

**Control: 1086 (56.5)**

**N = 675**

**Case: 342 (50.7)**

**Control: 333 (49.3)**

**OR: 1.33 (1.10-1.61)***

**Drinking status**

**No**

**Yes**

**No**

**Yes**

**History of T2DM**

**N = 1374**

**Case: 598 (42.9)**

**Control: 796 (57.1)**

**Reference**

**N = 529**

**Case: 239 (45.2)**

**Control: 290 (54.8)**

**OR: 1.10 (0.89-1.35)**

**No**

**N = 3781**

**Case: 1818 (48.1)**

**Control: 1963 (51.9)**

**Yes**

**N = 795**

**Case: 401 (50.4)**

**Control: 394 (49.6)**

**OR: 1.37 (1.14-1.64)***

**N = 388**

**Case: 238 (61.3)**

**Control: 150 (38.7)**

**No**

**Yes**

**Drinking status**

**N = 57**

**Case: 35 (61.4)**

**Control: 22 (38.6)**

**OR: 1.80 (1.02-3.17)***

**N = 99**

**Case: 74 (74.7)**

**Control: 25 (25.3)**

**OR: 3.73 (2.31-6.01)***

**Risk genotypes**

**1 + 2**

**0**

**N = 232**

**Case: 129 (55.6)**

**Control: 103 (44.4)**

**OR: 1.64 (1.22-2.18)***

**N = 156**

**Case: 109 (69.9)**

**Control: 47 (30.1)**

**No**

**Yes**

**History of T2DM**

**TN1**

**TN2**

**TN3**

**TN4**

**TN5**

**Figure S5 Associations of plasma MDA levels with SNP rs7212, No. of risk genotypes, and plasma TXNIP levels.** ANCOVA models were used to test the associations of plasma MDA levels with SNP rs7212 (a and d) and No. of risk genotypes (b and e) after adjusting for age, sex, BMI, smoking status, alcohol drinking status and histories of hypertension, hyperlipidemia and T2DM. The Pearson correlation test was used to assess the association of plasma MDA levels with plasma TXNIP levels (c and f).


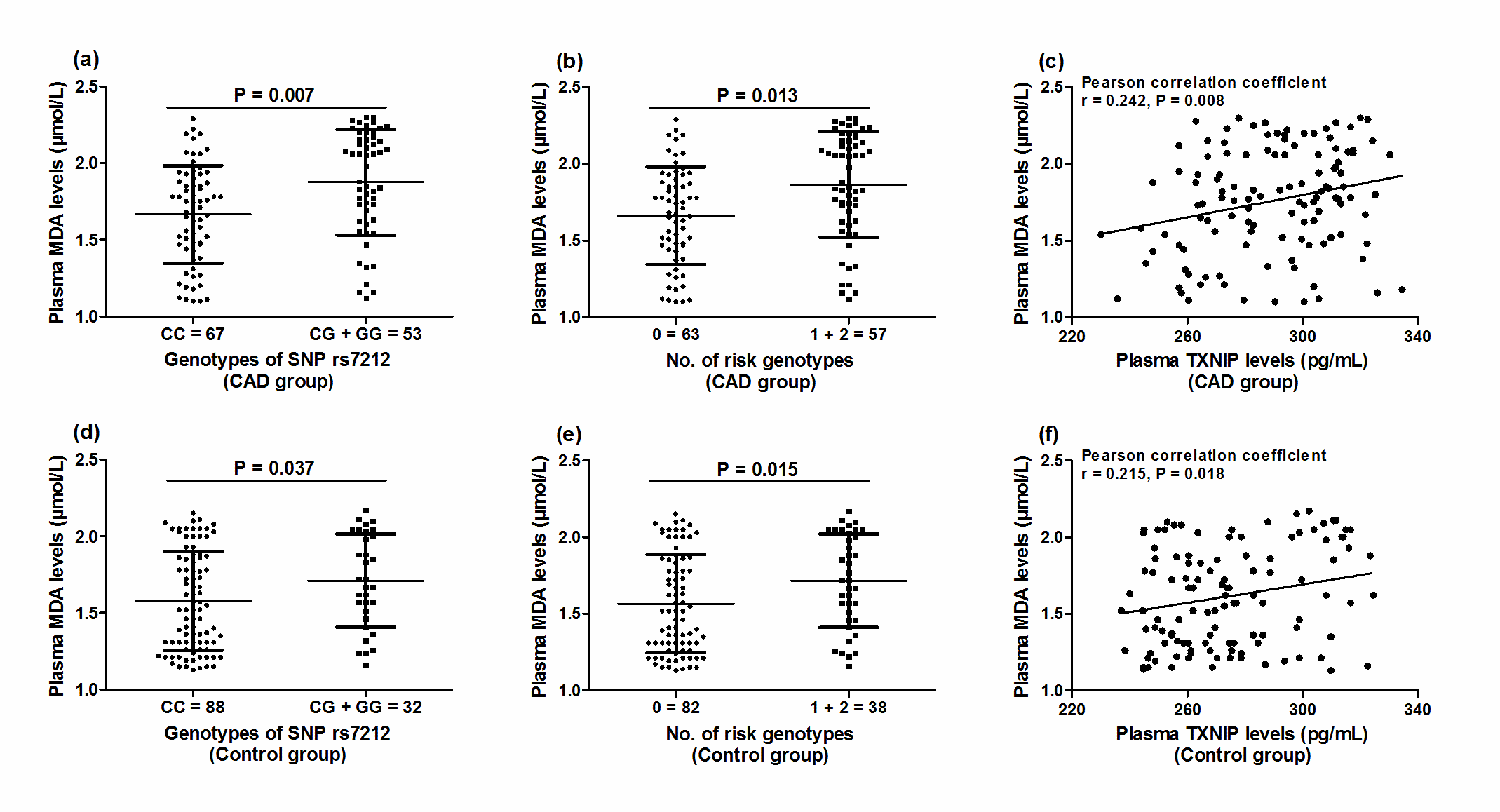


**Figure S6 Associations of methylation levels at cg19693031 with HbA1c (%), FPG, *TXNIP* mRNA expression, CAD risk and *TXNIP* SNPs.** The Pearson correlation test was used to assess the associations of methylation levels at cg19693031 with HbA1c (%) (a), FPG (b) and *TXNIP* mRNA expression (c). ANCOVA models were used to test the associations of methylation levels at cg19693031 with CAD risk (d) and *TXNIP* SNPs (e and f) after adjusting for age, sex, BMI, smoking status, alcohol drinking status and histories of hypertension, hyperlipidemia and T2DM.


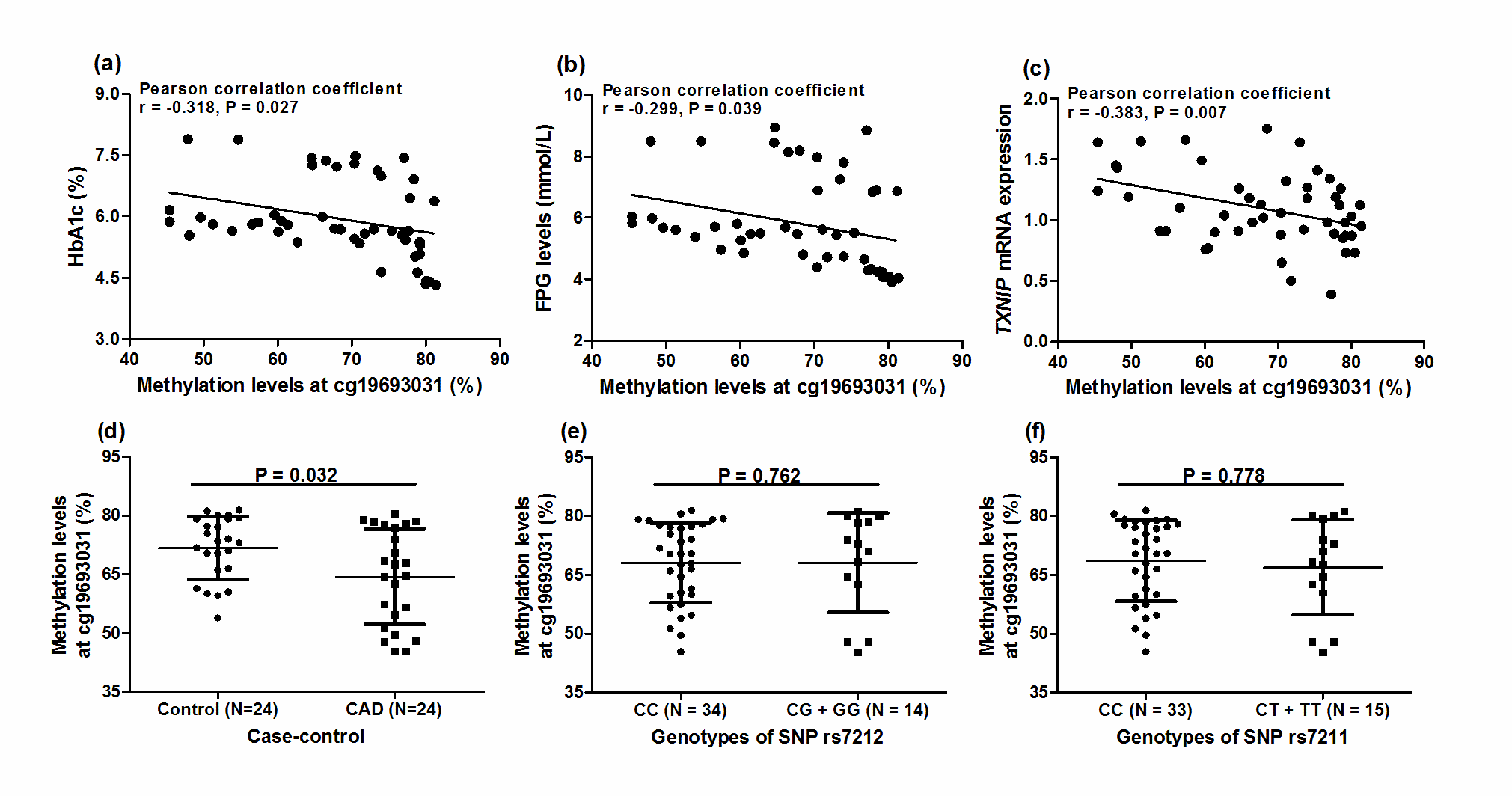

Supplement: Supplementary file 1 — Figure S1 HRM plots for different genotypes of three SNPs. Figure S2 Direct sequencing analyses for different genotypes of three SNPs. Figure S3 Analysis of the LD structure. Figure S4 Classification and regression tree for smoking status, alcohol drinking status, history of T2DM and the combined risk genotypes (SNPs rs7212+ rs7211) in all participants of our study. Figure S5 Associations of plasma MDA levels with SNP rs7212, No. of risk genotypes and plasma TXNIP levels. Figure S6 Associations of methylation levels at cg19693031 with HbA1c (%), FPG, TXNIP mRNA expression, CAD risk and TXNIP SNPs. Table S1 Characteristics of 3 SNPs in TXNIP gene. Table S2 Primer details and PCR conditions for HRM, direct sequencing and RT‐qPCR analyses in our study. Table S3 Clinical characteristics of participants in our study. Table S4 Associations of TXNIP SNPs with CAD risk in two sets of our study. Table S5 Associations of haplotypes and risk genotypes of SNP rs7212 and rs7211 with CAD risk in two sets of our study. Table S6 Stratification analyses of the combined risk genotypes (SNP rs7212+ rs7211) and CAD risk in our study. Table S7 Comparative analyses of clinical and genetic characteristics between the randomly selected participants and the whole samples. Table S8 Associations of TXNIP SNPs with TXNIP mRNA expression, plasma TXNIP and MDA levels. Data S1 Supplementary materials and methods. [file JCMM-20-2362-s001.docx]
